# Serum Metabolomic Profiling Across Five Oligoclonal Band (OCB) Patterns: A Targeted ^1^H-NMR Study in Serum

**DOI:** 10.3390/ijms27093904

**Published:** 2026-04-28

**Authors:** Pınar Şengül, Mustafa Serteser, Ahmet Tarik Baykal

**Affiliations:** 1 Department of Neuroscience, Graduate School of Health Sciences, Acibadem Mehmet Ali Aydinlar University, 34752 Istanbul, Turkey; pinar.sengul@live.acibadem.edu.tr; 2 Department of Medical Biochemistry, School of Medicine, Acibadem Mehmet Ali Aydinlar University, 34752 Istanbul, Turkey; mserteser@outlook.com; 3 Department of Biochemistry and Molecular Biology, Graduate School of Health Sciences, Acibadem Mehmet Ali Aydinlar University, 34752 Istanbul, Turkey; 4 Acibadem Labmed Clinical Laboratories, 34752 Istanbul, Turkey

**Keywords:** metabolomics, oligoclonal bands, ^1^H-NMR, neuroinflammation, serum biomarkers, amino acids, tricarboxylic acid cycle

## Abstract

Cerebrospinal fluid (CSF) oligoclonal band (OCB) analysis remains central to the diagnostic evaluation of neuroinflammatory diseases of the central nervous system (CNS), as it reflects intrathecal immunoglobulin synthesis. However, its reliance on lumbar puncture limits its applicability for screening and repeated longitudinal assessment. Serum metabolomics offers a minimally invasive strategy to explore peripheral biochemical correlates of central immune activity. Building on previous binary OCB comparisons, the present study extends serum metabolomic analysis to encompass all five classical OCB patterns, thereby capturing a broader immunological spectrum. A total of 92 adults undergoing diagnostic evaluation for suspected CNS inflammatory disorders were retrospectively stratified according to OCB type (Types 1–5). Serum samples were analysed using targeted ^1^H-NMR spectroscopy on a Bruker Avance Neo 600 MHz platform and processed using Bruker’s IVDr pipeline. Group-wise differences were assessed using non-parametric statistical testing with false discovery rate (FDR) correction, complemented by effect size estimation, exploratory multivariate analyses, and Receiver Operating Characteristic (ROC) modelling. Distributional characteristics were further examined using boxplots and violin plots. Across analytical approaches, several metabolites—most prominently leucine, 2-oxoglutaric acid, histidine, threonine, and glycerol—exhibited nominal variation and moderate effect sizes across OCB patterns. Rather than discrete metabolic separation, these metabolites demonstrated graded shifts in central tendency accompanied by substantial overlap between groups. Unsupervised principal component analysis did not reveal robust clustering, while supervised multivariate models highlighted amino acid- and tricarboxylic acid cycle-related metabolites as contributors to partial differentiation. Post hoc power analysis indicated limited sensitivity to detect small-to-moderate effects under multiple-testing correction, supporting an exploratory interpretation of the findings. Taken together, this first targeted serum ^1^H-NMR metabolomic evaluation spanning all classical OCB patterns suggests that peripheral metabolic profiles may reflect graded immunometabolic variation associated with intrathecal immune activity. While not intended for diagnostic classification, these findings provide a spectrum-based framework for integrating serum metabolomics with OCB phenotyping and identify candidate metabolites for future prospectively powered and clinically characterised studies.

## 1. Introduction

Chronic neuroinflammatory diseases of the central nervous system (CNS) comprise a heterogeneous group of disorders characterised by immune-mediated demyelination, neurodegeneration, and compartmentalised humoral immune responses [1]. Within this spectrum, the detection of cerebrospinal fluid (CSF) immunoglobulin G (IgG) oligoclonal bands (OCBs) remains a cornerstone laboratory marker of intrathecal IgG synthesis. Although most commonly associated with multiple sclerosis (MS), OCBs are also observed in neuromyelitis optica spectrum disorder (NMOSD), neurosarcoidosis, neuro-Behçet’s disease, and various infectious or autoimmune CNS conditions, highlighting that OCB positivity reflects immune activation rather than disease-specific pathology [2,3].

OCB detection is routinely performed using isoelectric focusing followed by immunoblotting and provides important diagnostic support in clinical practice. However, reliance on lumbar puncture limits its suitability for population screening, repeated longitudinal monitoring, and large-scale biomarker studies [1]. Recent diagnostic revisions, including updates to the McDonald criteria, have expanded the biological framework of MS by incorporating the κ-free light chain (κ-FLC) index as an alternative marker of intrathecal immunoglobulin synthesis [4,5]. These developments underscore the growing interest in quantifiable and standardisable biomarkers that may complement CSF-based measures and provide additional insight into central immune activity, particularly in early or diagnostically ambiguous cases.

In this context, metabolomics has emerged as a promising analytical approach for capturing systemic biochemical alterations associated with neuroinflammation and neurodegeneration [6,7]. Metabolomic profiling of serum and CSF has revealed perturbations in amino acid metabolism, tricarboxylic acid (TCA) cycle intermediates, lipid turnover, and oxidative stress pathways in MS and related disorders [8]. Amino acids such as leucine, histidine, and glycine are known to participate in immunometabolic processes: leucine influences mTOR-dependent immune cell metabolism; histidine, via histamine, modulates microglial activity and neuronal excitability; and glycine contributes to redox homeostasis through glutathione synthesis [9,10]. Similarly, TCA cycle intermediates, including succinate and 2-oxoglutarate, have been implicated in macrophage polarisation and inflammatory signalling [11]. Together, these metabolites form a biochemical interface linking systemic metabolism with immune activation.

Despite these advances, most metabolomic studies in neuroinflammatory conditions have relied on binary classifications, such as OCB-positive versus OCB-negative status or disease-based groupings. While informative, such approaches may oversimplify the biological heterogeneity of intrathecal immune responses. The classical five-type OCB classification captures a broader immunophenotypic continuum, ranging from absence of intrathecal synthesis (Type 1), through varying degrees of intrathecal IgG production with or without systemic components (Types 2–4), to monoclonal gammopathy patterns (Type 5) [3]. Examining metabolic variation across this full spectrum may therefore provide a more nuanced understanding of peripheral correlates of intrathecal immune activity.

In previous work, our group applied targeted ^1^H-NMR serum metabolomics to a binary comparison of OCB Type 1 and Type 2 profiles and identified leucine as a metabolite associated with intrathecal IgG synthesis [12]. However, that analysis was limited to two OCB categories and did not address the broader diversity of OCB phenotypes. Whether peripheral metabolite profiles vary in a graded manner across all five OCB patterns remains unclear.

Accordingly, the present study applies targeted ^1^H-NMR spectroscopy to serum samples from 92 adults undergoing diagnostic evaluation for suspected CNS inflammatory disorders in Türkiye, stratified according to OCB Types 1–5. By integrating non-parametric statistical testing, effect size estimation, exploratory multivariate analyses, and distributional visualisation, this work aims to characterise serum metabolic variation across the OCB spectrum. Rather than proposing diagnostic biomarkers, the objective is to explore whether amino acid- and TCA cycle-related metabolites exhibit graded associations with OCB-defined intrathecal immune activity and to identify candidate metabolites for future prospectively powered and clinically characterised studies.

## 2. Results

### 2.1. Participant Demographics and Laboratory Characteristics

A total of 92 adults undergoing diagnostic evaluation for suspected central nervous system (CNS) inflammatory disorders were included in the study and stratified according to oligoclonal band (OCB) type (Types 1–5). Demographic variables and routine laboratory parameters are summarised in Table 1.

Significant differences were observed across OCB subgroups with respect to age, while sex distribution did not differ significantly between groups.

Routine biochemical parameters, including serum glucose, creatinine, liver enzymes, and inflammatory markers, were comparable across groups. These findings indicate that the OCB subgroups were broadly similar with respect to baseline demographic and laboratory characteristics, supporting the validity of subsequent metabolomic comparisons.

### 2.2. Univariate Metabolite Comparisons Across OCB Types

All quantified serum metabolites demonstrated non-Gaussian distributions and were therefore analysed using non-parametric methods. Group-wise comparisons across OCB Types 1–5 were performed using the Kruskal–Wallis test with false discovery rate (FDR) correction. Summary statistics for all metabolites are provided in Table 2, and detailed pairwise Dunn post hoc comparisons are presented in Table S1.

Several metabolites exhibited nominal group-wise variation across OCB patterns. Leucine and 2-oxoglutaric acid showed the lowest unadjusted *p*-values, with additional metabolites including histidine, threonine, citric acid, and glycerol demonstrating nominal differences. Following FDR correction, no comparison reached the conventional significance threshold (q < 0.05); therefore, all findings are interpreted descriptively within an exploratory framework.

Pairwise analyses indicated that differences were most pronounced between OCB Type 1 and Type 2 for leucine and 2-oxoglutaric acid, while comparisons involving OCB Types 3–5 showed increased dispersion and heterogeneity.

### 2.3. Distribution of Individual Serum Metabolites by OCB Group

Boxplots illustrating the distribution of selected serum metabolites across oligoclonal band (OCB) Types 1–5. Metabolites shown include leucine, 2-oxoglutaric acid, histidine, threonine, and glycerol. Boxes represent interquartile ranges with median values indicated, while whiskers denote variability outside the upper and lower quartiles. Substantial overlap between OCB groups is observed, consistent with graded rather than discrete metabolic variation.

### 2.4. Distributional Visualisation of Serum Metabolites

**Figure 1:**
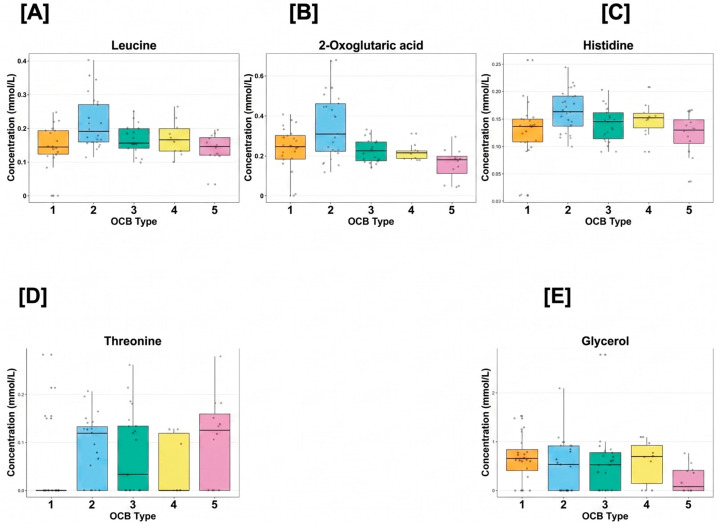
Boxplots of key serum metabolites across OCB types. (**A**) Leucine, (**B**) 2-oxoglutaric acid, (**C**) histidine, (**D**) threonine, and (**E**) glycerol.

To visualise group-wise metabolite distributions, boxplots were generated for selected metabolites across OCB Types 1–5 (Figure 1). These plots illustrate medians, interquartile ranges, and outliers, allowing comparison of central tendency and variability across groups.

Leucine and 2-oxoglutaric acid displayed higher median concentrations in OCB Type 2 compared with Type 1, with partially overlapping distributions across other OCB patterns. Histidine and threonine showed broader distributions with substantial overlap between all OCB types, while glycerol demonstrated marked inter-individual variability without a consistent directional trend.

Complementary violin plots illustrating full distributional density are provided in the Supplementary Materials (Figures S1–S5).

To explore global variance structure in the serum metabolomic dataset, unsupervised principal component analysis (PCA) was performed. PCA did not reveal distinct clustering according to OCB type, indicating substantial inter-individual variability.

### 2.5. Principal Component Analysis

**Figure 2:**
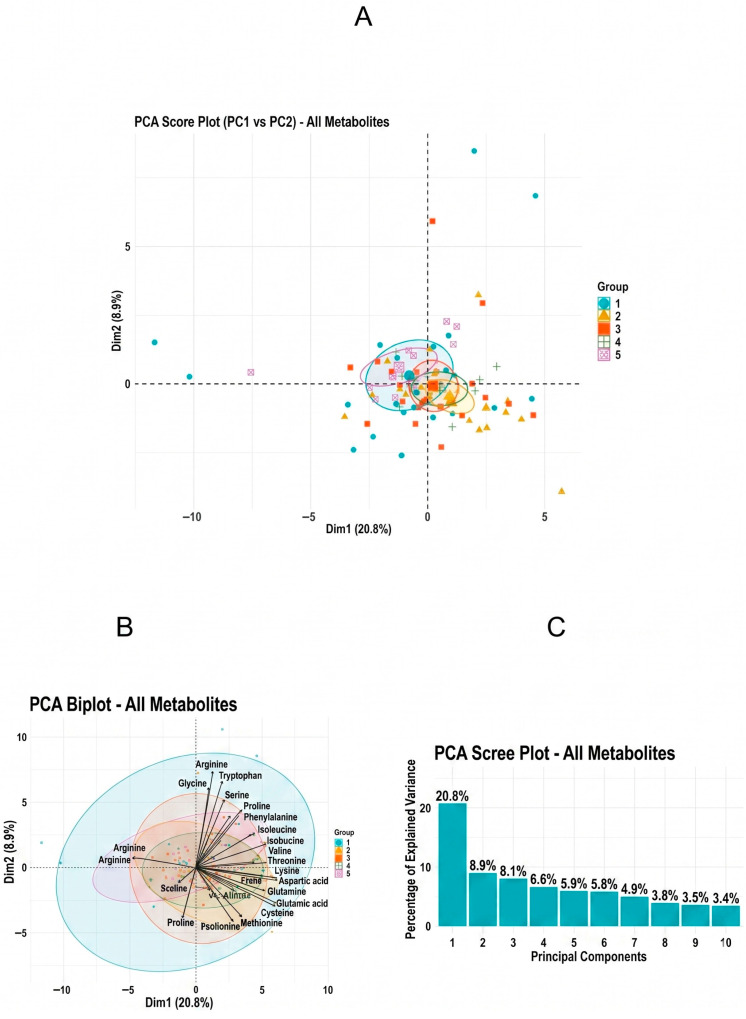
Principal component analysis (PCA) of serum metabolomic profiles across OCB types. (**A**) PCA score plot, (**B**) loading plot, and (**C**) scree plot showing the variance explained by principal components.

Unsupervised principal component analysis (PCA) was conducted to assess the global variance structure of the serum metabolomic dataset. The first two principal components explained a limited proportion of total variance. As shown in Figure 2, samples did not cluster distinctly according to OCB type, with substantial overlap observed across all subgroups.

These findings indicate that between-group differences were modest relative to overall biological variability. PCA was therefore used as a descriptive tool rather than as evidence of unsupervised group separation.

**Figure 3:**
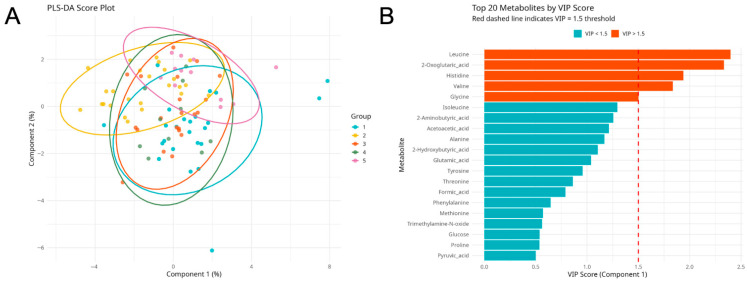
Partial least squares discriminant analysis (PLS-DA) of serum metabolomic profiles across OCB types. (**A**) PLS-DA score plot showing group separation and (**B**) variable importance in projection (VIP) scores of the top 20 metabolites contributing most strongly to group discrimination.

Partial least squares discriminant analysis (PLS-DA) was applied as an exploratory supervised approach to identify metabolites contributing to group differentiation (Figure 3A). Partial separation between OCB groups was observed, with substantial overlap across classes. Variable importance in projection (VIP) scores were calculated to identify metabolites contributing most strongly to model components (Figure 3B).

Partial least squares discriminant analysis (PLS-DA) was applied as an exploratory supervised approach to identify metabolites contributing to differentiation across OCB patterns. The PLS-DA score plot demonstrated partial separation between selected OCB groups, although marked overlap persisted.

Variable importance in projection (VIP) analysis identified leucine, 2-oxoglutaric acid, histidine, valine, and glycine as metabolites contributing most strongly to the model. Given the overlap between groups and limited sample sizes within individual OCB categories, PLS-DA results are interpreted as exploratory and not indicative of robust classification.

Receiver Operating Characteristic (ROC) analysis was performed to explore the discriminatory performance of selected metabolites and metabolite combinations. ROC curves were derived from internally constructed logistic regression models and are interpreted for exploratory purposes only.

### 2.6. Effect Size Estimation and Statistical Power

To complement *p*-value-based inference, effect sizes for pairwise group comparisons were estimated using Cliff’s delta. Several metabolites demonstrated moderate effect sizes, particularly in comparisons involving OCB Type 2 relative to Type 1. Effect size estimates are summarised in Table S2.

A post hoc power analysis was performed under the same multiple-testing framework applied to the primary analyses. Power estimates indicated limited sensitivity to detect small-to-moderate effects and incomplete sensitivity even for larger effects, given the current group sizes. Power curves and required sample size estimates are provided in the Supplementary Materials (Figure S6).

### 2.7. Receiver Operating Characteristic Analysis

Receiver Operating Characteristic (ROC) analysis was conducted to explore the discriminatory performance of selected metabolites and metabolite combinations. Single-metabolite ROC curves yielded modest area-under-the-curve (AUC) values, while a multimetabolite logistic model incorporating leucine, histidine, and citric acid demonstrated improved internal performance.

**Figure 4:**
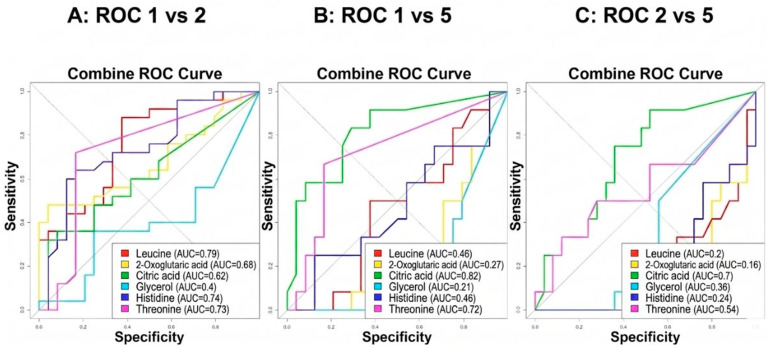
Receiver Operating Characteristic (ROC) curves of selected metabolites and metabolite combinations for discrimination between OCB types. ROC curves were derived from internally constructed models and are presented for exploratory purposes.

ROC curves for individual metabolites and the combined model are shown in Figure 4. These results represent internally derived exploratory performance estimates and were not externally validated.

### 2.8. Supplementary Analyses

Additional visualisations of metabolite distributions, post hoc power analyses, and Random Forest modelling results are provided in the Supplementary Materials (Figures S1–S8). These analyses further contextualise the observed univariate and multivariate findings but are not intended to support inferential conclusions.

## 3. Discussion

This study extends previous two-group serum metabolomic analyses by incorporating all five established oligoclonal band (OCB) phenotypes into a comprehensive, spectrum-based comparison. Using targeted ^1^H-NMR spectroscopy, we observed graded metabolic variation across OCB types in individuals undergoing diagnostic evaluation for suspected central nervous system (CNS) inflammatory disorders. These findings support the concept that peripheral metabolic profiles may reflect differing degrees of intrathecal immune activity, rather than discrete diagnostic states [3].

Across univariate and multivariate analyses, a coherent pattern emerged dominated by amino acid- and tricarboxylic acid (TCA) cycle-related metabolites. Leucine, 2-oxoglutaric acid, histidine, valine, and citric acid consistently contributed to variation across OCB patterns. The prominence of branched-chain amino acids (BCAAs) and TCA intermediates aligns with growing evidence that immune activation is tightly coupled to cellular bioenergetics and metabolic adaptations associated with immune activation [13,14]. Rather than indicating disease-specific signatures, these metabolites likely reflect an immunometabolic continuum accompanying intrathecal immune activation.

Leucine again emerged as a key metabolite associated with OCB-defined immune activity, consistent with its previously reported elevation in OCB Type 2 serum and its established role as an mTOR-dependent regulator of T-cell metabolism and glial responses [15,16]. Similarly, elevated 2-oxoglutaric acid may reflect enhanced anaplerotic flux through the TCA cycle, supporting lymphocyte proliferation and macrophage polarisation during immune activation [17,18]. Histidine, a precursor of histamine involved in microglial modulation and neuroimmune signalling, also showed consistent contributions across analytical approaches, suggesting a potential link between systemic histidine metabolism and CNS immune tone [9,10].

Interestingly, citric acid and glycerol demonstrated variation, particularly involving OCB Type 5, a pattern associated with systemic rather than CNS-restricted immunoglobulin production. This observation may reflect metabolic states linked to systemic immune activation or monoclonal gammopathies, which have previously been associated with altered citrate and glycerol turnover [8,19]. However, given the limited sample size within this subgroup, these findings should be interpreted cautiously.

Unsupervised and supervised multivariate analyses further supported a graded rather than categorical interpretation. Principal component analysis did not reveal distinct clustering by OCB type, while partial least squares discriminant analysis captured modest separation accompanied by substantial overlap. This pattern underscores that metabolic transitions occur along a continuum rather than at discrete immunological boundaries [20]. Although Receiver Operating Characteristic analyses suggested improved internal performance for selected multimetabolite combinations, these results were derived within the same cohort and were not externally validated, and thus should be regarded as exploratory [21].

Several limitations warrant consideration. The cross-sectional and retrospective design precludes causal inference, and unmeasured factors such as nutritional status or prior treatment exposure may influence serum metabolite levels. In addition, κ-free light chain (κ-FLC) measurements—now incorporated into updated MS diagnostic criteria—were unavailable in this cohort [4,5]. Integrating longitudinal metabolomics with κ-FLC and other CSF-based biomarkers will be essential to clarify the temporal dynamics and clinical relevance of the observed metabolic patterns.

## 4. Materials and Methods

### 4.1. Study Design and Cohort Selection

This retrospective, observational study was conducted using archived serum samples collected during routine diagnostic evaluation for suspected central nervous system (CNS) inflammatory disorders. All samples originated from individuals who underwent cerebrospinal fluid (CSF) and paired serum analysis for oligoclonal band (OCB) testing as part of standard clinical work-up. Residual serum samples remaining after routine analyses were subsequently utilised for targeted metabolomic profiling.

A total of 92 adult participants were included. Individuals were stratified according to classical OCB patterns (Types 1–5) based on paired CSF and serum immunoglobulin G (IgG) electrophoretic profiles. As the study was based on retrospectively collected biobank material, detailed clinical diagnoses, disease duration, disability scores, and treatment status at the time of sampling were not uniformly available and were therefore not included in the analyses.

### 4.2. Inclusion and Exclusion Criteria

Inclusion criteria comprised adult individuals (≥18 years) who underwent routine diagnostic evaluation for suspected central nervous system (CNS) inflammatory disorders and for whom paired cerebrospinal fluid (CSF) and serum samples were available for oligoclonal band (OCB) analysis. Only participants with a clearly defined classical OCB pattern (Types 1–5), as determined by paired CSF–serum electrophoretic analysis, were included.

Exclusion criteria comprised incomplete or uninterpretable OCB results, insufficient residual serum volume for ^1^H-NMR analysis, poor spectral quality failing IVDr quality control criteria, and samples subjected to repeated freeze–thaw cycles. Due to the retrospective biobank-based design, no additional exclusion was applied based on clinical diagnosis, disease duration, disability status, or treatment exposure, as these data were not uniformly available.

### 4.3. Oligoclonal Band Analysis

OCB analysis was performed in routine clinical practice using isoelectric focusing followed by immunoblotting (Hydragel 9 CSF Isofocusing, Sebia, Lisses, France), in accordance with established laboratory protocols. Paired CSF and serum samples were analysed simultaneously to determine intrathecal IgG synthesis patterns. OCB results were classified into five classical patterns as previously described: Type 1 (no OCBs), Type 2 (CSF-restricted OCBs), Type 3 (CSF-restricted OCBs with additional identical bands in serum), Type 4 (identical CSF and serum bands), and Type 5 (monoclonal gammopathy pattern). OCB classification was performed independently of metabolomic analysis.

### 4.4. Serum Sample Handling and Preparation

Serum samples were stored at −80 °C until analysis and underwent no more than one freeze–thaw cycle prior to metabolomic measurement. Sample preparation followed the standardised Bruker IVDr protocol for serum metabolomics (Bruker BioSpin GmbH, Rheinstetten, Germany). Briefly, serum aliquots were mixed with IVDr buffer containing deuterated water and internal reference compounds, vortexed, and transferred to 5 mm NMR tubes. All samples were prepared in a randomised order to minimise batch effects.

### 4.5. ^1^H-NMR Spectroscopy

Targeted ^1^H-nuclear magnetic resonance (^1^H-NMR) spectroscopy was performed using a Bruker Avance Neo 600 MHz spectrometer (Bruker BioSpin GmbH, Rheinstetten, Germany) equipped with a temperature-controlled probe. Spectra were acquired at 310 K using standard one-dimensional pulse sequences optimised for serum metabolomics. A fixed number of scans and relaxation delay were applied across all samples to ensure quantitative consistency.

Spectral acquisition, processing, and metabolite quantification were performed using TopSpin software (version 4.5, Bruker BioSpin GmbH, Rheinstetten, Germany) within the Bruker IVDr platform (Bruker BioSpin GmbH, Rheinstetten, Germany), which provides automated spectral alignment, baseline correction, and absolute metabolite quantification based on internal calibration, consistent with established NMR metabolomics protocols [22]. Only metabolites passing quality control criteria specified by the IVDr workflow were included in downstream analyses.

### 4.6. Metabolite Selection and Data Preprocessing

Quantified metabolites were log-transformed where appropriate to reduce skewness and improve comparability across samples. Prior to statistical analysis, metabolite concentrations were inspected for outliers and missing values. Metabolites with excessive missingness or unreliable quantification were excluded. No imputation was applied for missing values.

### 4.7. Statistical Analysis

All statistical analyses were performed using R software (version 4.3.1, R Foundation for Statistical Computing, Vienna, Austria), implemented in RStudio (version 2025.05.0+496, Posit Software, Boston, MA, USA). Data distributions were assessed using the Shapiro–Wilk test and visual inspection. As metabolite concentrations exhibited non-normal distributions, non-parametric statistical methods were applied throughout.

Group-wise comparisons across OCB Types 1–5 were conducted using the Kruskal–Wallis test. Where applicable, pairwise comparisons were performed using Dunn’s post hoc test. To control for multiple testing, false discovery rate (FDR) correction was applied using the Benjamini–Hochberg procedure. Statistical significance was defined as q < 0.05; however, nominal associations are also reported descriptively in accordance with the exploratory nature of the study.

Effect sizes for pairwise comparisons were estimated using Cliff’s delta to provide magnitude-based interpretation independent of *p*-values.

### 4.8. Multivariate Analyses

Unsupervised principal component analysis (PCA) was performed to explore global variance structure within the metabolomic dataset. Prior to PCA, metabolite concentrations were centred and scaled.

Partial least squares discriminant analysis (PLS-DA) was applied as an exploratory supervised method to identify metabolites contributing to group differentiation. Model performance was assessed descriptively, and variable importance in projection (VIP) scores were calculated to identify metabolites contributing most strongly to model components. Given the limited sample size and potential for overfitting, PLS-DA results were interpreted cautiously.

Random Forest modelling was conducted as an additional exploratory, non-linear approach. Variable importance measures were extracted to identify metabolites contributing to model performance. Random Forest analyses are reported in the Supplementary Material.

### 4.9. Receiver Operating Characteristic Analysis

Receiver Operating Characteristic (ROC) analysis was performed to explore the discriminatory performance of selected metabolites and metabolite combinations. Logistic regression models were constructed using selected metabolites identified in univariate and multivariate analyses. Area-under-the-curve (AUC) values were calculated as internal performance estimates following standard ROC methodology [23]. No external validation cohort was available, and ROC analyses are therefore interpreted as exploratory.

### 4.10. Power Analysis

Post hoc power analysis was conducted to assess statistical sensitivity under the applied multiple-testing framework. Power was estimated as a function of observed effect sizes and current group sample sizes. Power curves and estimated sample sizes required to achieve 80% power are presented in the Supplementary Material. These analyses were used to contextualise non-significant findings rather than to inform inferential conclusions.

### 4.11. Ethical Considerations

This study was conducted in accordance with the principles of the Declaration of Helsinki. Ethical approval was obtained from the Acıbadem Mehmet Ali Aydınlar University Medical Research Evaluation Committee (ATADEK). The study protocol entitled “Investigation of changes in levels of small molecules identified by NMR-based quantitative metabolomic analysis in cerebrospinal fluid and serum in multiple sclerosis” was reviewed and approved at the ATADEK meeting held on 10 March 2023, with the decision number 2023/4-132.

The study was classified as ethically appropriate by the committee. As this research was based on retrospectively collected and anonymised biobank material obtained during routine clinical procedures, the requirement for informed consent was waived in accordance with institutional and national regulations. No additional interventions or sample collection procedures were performed for the purposes of this study.

## 5. Conclusions

This study provides a targeted ^1^H-NMR-based serum metabolomic evaluation across all five classical oligoclonal band (OCB) patterns in individuals undergoing diagnostic assessment for suspected central nervous system (CNS) inflammatory disorders. By extending beyond binary OCB classifications, the present work adopts a spectrum-based approach to explore peripheral metabolic variation in relation to OCB-defined intrathecal immune activity.

Across univariate, multivariate, and distributional analyses, several metabolites—particularly amino acids and tricarboxylic acid (TCA) cycle-related intermediates—showed graded variation across OCB patterns. Rather than discrete metabolic separation, these findings were characterised by modest effect sizes, substantial overlap between groups, and heterogeneous distributions, consistent with the interpretation of OCB phenotypes as reflecting a continuum of immunological states.

Given the retrospective design, limited and unbalanced subgroup sizes, and restricted statistical power after multiple-testing correction, the findings should be interpreted as exploratory and hypothesis-generating. Given the unequal group sizes and multiple-testing correction, the study was primarily powered to detect moderate-to-large effects, and smaller effect sizes may have remained undetected. Importantly, the absence of statistically significant differences following correction does not preclude biological relevance but underscores the need for adequately powered, prospectively designed studies.

While serum metabolomics is not proposed as a diagnostic alternative to CSF-based OCB analysis, the present results demonstrate the feasibility of integrating standardised serum metabolomic profiling with OCB phenotyping to investigate peripheral correlates of intrathecal immune processes. The metabolites highlighted in this study represent biologically plausible candidates for future investigation.

In summary, this work delineates the scope and limitations of detectable serum metabolic variation across the OCB spectrum and provides a methodologically transparent foundation for future studies combining metabolomics with clinical, immunological, and longitudinal data to further elucidate the immunometabolic landscape of neuroinflammatory conditions.

## Figures and Tables

**Table 1:** Demographic and laboratory characteristics of participants stratified by OCB type.

Variable	Type 1	Type 2	Type 3	Type 4	Type 5	*p*-Value
Sample Size (*n*)	24	25	22	11	12	-
Age (years)	40.50 (29.25–56.75)	30.00 (23.00–36.00)	45.00 (37.00–50.00)	61.00 (52.00–71.50)	68.00 (58.25–73.25)	4.49 × 10^−7^
IgG Index	0.49 (0.46–0.54)	0.78 (0.67–1.12)	0.90 (0.76–1.24)	0.59 (0.53–0.61)	0.51 (0.48–0.58)	5.62 × 10^−14^
IgG CSF (mg/L)	2.95 (2.15–3.60)	3.60 (2.65–4.30)	9.55 (7.27–23.00)	4.94 (2.68–6.68)	4.12 (2.69–5.45)	1.53 × 10^−7^
IgG Serum (mg/dL)	1091.00 (948.75–1201.25)	1122.00 (1002.00–1250.00)	953.00 (882.00–1256.00)	951.00 (766.50–1034.50)	1111.00 (901.75–1294.25)	0.2234
IgG CSF/Serum Ratio	2.65 (2.10–3.19)	3.61 (2.79–4.37)	9.97 (7.12–16.85)	4.78 (3.08–8.75)	3.42 (2.52–4.92)	1.51 × 10^−8^
Albumin CSF (mg/L)	19.60 (16.30–23.07)	15.90 (12.30–21.80)	39.40 (35.00–48.20)	35.00 (19.25–42.10)	26.15 (18.95–31.52)	3.18 × 10^−7^
Albumin Serum (mg/dL)	3685.00 (3460.00–3895.00)	4100.00 (3960.00–4380.00)	3860.00 (3440.00–3950.00)	3520.00 (2980.00–3925.00)	3765.00 (3497.50–3970.00)	9.43 × 10^−4^
Albumin Ratio (×10^−3^)	5.20 (4.45–6.31)	3.97 (3.07–5.51)	10.12 (9.07–12.43)	11.11 (4.94–15.82)	7.35 (4.83–9.18)	3.84 × 10^−8^
Gender (Female), *n* (%)	15 (62.5%)	17 (68.0%)	11 (50.0%)	3 (27.3%)	4 (33.3%)	0.1115

CSF measurements are reported in mg/L and serum measurements in mg/dL, consistent with standard clinical laboratory reporting.

**Table 2:** Distribution of serum metabolite concentrations (mmol/L) across OCB types.

Metabolite (mmol/L)	Type 1 (*n* = 24)	Type 2 (*n* = 25)	Type 3 (*n* = 21)	Type 4 (*n* = 10)	Type 5 (*n* = 12)
Leucine	0.145 (0.123–0.194)	0.191 (0.159–0.271)	0.157 (0.141–0.200)	0.167 (0.133–0.199)	0.147 (0.121–0.173)
2-Oxoglutaric acid	0.246 (0.184–0.302)	0.308 (0.222–0.461)	0.225 (0.175–0.268)	0.214 (0.186–0.225)	0.181 (0.113–0.198)
Citric acid	0.015 (0.000–0.082)	0.056 (0.000–0.169)	0.092 (0.000–0.162)	0.045 (0.000–0.151)	0.158 (0.082–0.193)
Glycerol	0.658 (0.413–0.837)	0.533 (0.000–0.915)	0.527 (0.000–0.776)	0.695 (0.149–0.928)	0.081 (0.000–0.410)
Histidine	0.137 (0.108–0.149)	0.163 (0.137–0.192)	0.145 (0.114–0.162)	0.152 (0.134–0.161)	0.130 (0.105–0.149)
Threonine	0.000 (0.000–0.000)	0.119 (0.000–0.133)	0.033 (0.000–0.134)	0.000 (0.000–0.119)	0.125 (0.000–0.159)

## Data Availability

The data presented in this study are available from the corresponding author upon reasonable request. The data are not publicly available due to ethical and privacy restrictions associated with patient-related biobank material.

## Supplementary Materials

The following supporting information can be downloaded at: https://www.mdpi.com/article/10.3390/ijms27093904/s1.
