# Supplementary material for: Serum Metabolomic Profiling Across Five Oligoclonal Band (OCB) Patterns: A Targeted 1H-NMR Study in Serum
Source: Int J Mol Sci. 2026 Apr 28;27(9):3904. doi: 10.3390/ijms27093904 (PMC13163819; doi:10.3390/ijms27093904)
Supplement: Supplementary file 1 [file ijms-27-03904-s001.zip › ijms-4050794-supplementary.pdf]

Supplementary Materials- IJMS- 4050794

Table S1. Detailed pairwise Dunn post-hoc comparisons of serum metabolite concentrations across OCB types.

Metabolite	Group 1	Group 2	n1	n2	Test statistic	P-value	Adjusted P-value (FDR)	Significance
2-Oxoglutaric acid	Type 1	Type 2	24	25	1.804310738738	0.0712	0.1389	ns
2-Oxoglutaric acid	Type 1	Type 3	24	21	-0.725551097206225	0.4681	0.5201	ns
2-Oxoglutaric acid	Type 1	Type 4	24	10	-0.72763018162945	0.4668	0.5201	ns
2-Oxoglutaric acid	Type 1	Type 5	24	12	-2.3856576093513	0.0170	0.0568	ns
2-Oxoglutaric acid	Type 2	Type 3	25	21	-2.47436439691753	0.0133	0.0568	ns
2-Oxoglutaric acid	Type 2	Type 4	25	10	-2.11001358913212	0.0349	0.0871	ns

2-Oxoglutaric acid	Type 2	Type 5	25	12	-3.86995086584417	1E-04	0.0011	**
2-Oxoglutaric acid	Type 3	Type 4	21	10	-0.148538196867261	0.8819	0.8819	ns
2-Oxoglutaric acid	Type 3	Type 5	21	12	-1.73170347293547	0.0833	0.1389	ns
2-Oxoglutaric acid	Type 4	Type 5	10	12	-1.33026930127829	0.1834	0.2620	ns
Citric acid	Type 1	Type 2	24	25	1.42608429740253	0.1538	0.2564	ns
Citric acid	Type 1	Type 3	24	21	1.61352522530055	0.1066	0.2133	ns
Citric acid	Type 1	Type 4	24	10	0.739823854865532	0.4594	0.6563	ns
Citric acid	Type 1	Type 5	24	12	3.07840116549105	0.0021	0.0208	*
Citric acid	Type 2	Type 3	25	21	0.2520069153328	0.8010	0.8010	ns

Citric acid	Type 2	Type 4	25	10	-0.344975333937128	0.7301	0.8010	ns
Citric acid	Type 2	Type 5	25	12	1.93867884833707	0.0525	0.1952	ns
Citric acid	Type 3	Type 4	21	10	-0.530106207323784	0.5960	0.7450	ns
Citric acid	Type 3	Type 5	21	12	1.67529925626982	0.0939	0.2133	ns
Citric acid	Type 4	Type 5	10	12	1.89156462949973	0.0585	0.1952	ns
Glycerol	Type 1	Type 2	24	25	-1.37141757973116	0.1702	0.3405	ns
Glycerol	Type 1	Type 3	24	21	-1.24891341277667	0.2117	0.3528	ns
Glycerol	Type 1	Type 4	24	10	-0.214757843531622	0.8300	0.9222	ns
Glycerol	Type 1	Type 5	24	12	-2.75750537461823	0.0058	0.0582	ns

Glycerol	Type 2	Type 3	25	21	0.0632791606908397	0.9495	0.9495	ns
Glycerol	Type 2	Type 4	25	10	0.831405096102772	0.4057	0.5584	ns
Glycerol	Type 2	Type 5	25	12	-1.66010625656178	0.0969	0.2422	ns
Glycerol	Type 3	Type 4	21	10	0.760913661597914	0.4467	0.5584	ns
Glycerol	Type 3	Type 5	21	12	-1.66284966179418	0.0963	0.2422	ns
Glycerol	Type 4	Type 5	10	12	-2.08815244142276	0.0368	0.1839	ns
Histidine	Type 1	Type 2	24	25	3.0706319844104	0.0021	0.0214	*
Histidine	Type 1	Type 3	24	21	0.861768278038283	0.3888	0.4860	ns
Histidine	Type 1	Type 4	24	10	1.43877600098951	0.1502	0.3004	ns
Histidine	Type 1	Type 5	24	12	-0.242775708924707	0.8082	0.8082	ns

Histidine	Type 2	Type 3	25	21	-2.09457051654513	0.0362	0.1207	ns
Histidine	Type 2	Type 4	25	10	-0.897919110722206	0.3692	0.4860	ns
Histidine	Type 2	Type 5	25	12	-2.74308660412824	0.0061	0.0304	*
Histidine	Type 3	Type 4	21	10	0.739260899548012	0.4597	0.5108	ns
Histidine	Type 3	Type 5	21	12	-0.948775648055488	0.3427	0.4860	ns
Histidine	Type 4	Type 5	10	12	-1.46522015384652	0.1429	0.3004	ns
Leucine	Type 1	Type 2	24	25	3.34421705830422	8E-04	0.0083	**
Leucine	Type 1	Type 3	24	21	1.10313375634791	0.2700	0.4037	ns
Leucine	Type 1	Type 4	24	10	0.855384279702088	0.3923	0.4904	ns

Leucine	Type 1	Type 5	24	12	-0.390646680613892	0.6961	0.7734	ns
Leucine	Type 2	Type 3	25	21	-2.11504893016711	0.0344	0.1148	ns
Leucine	Type 2	Type 4	25	10	-1.6937271795023	0.0903	0.2258	ns
Leucine	Type 2	Type 5	25	12	-3.11457868303758	0.0018	0.0092	**
Leucine	Type 3	Type 4	21	10	-0.019961127892127	0.9841	0.9841	ns
Leucine	Type 3	Type 5	21	12	-1.29254781693368	0.1962	0.3923	ns
Leucine	Type 4	Type 5	10	12	-1.07449063836574	0.2826	0.4037	ns
Threonine	Type 1	Type 2	24	25	2.84034688358237	0.0045	0.0397	*
Threonine	Type 1	Type 3	24	21	1.98263972201818	0.0474	0.1580	ns

Threonine	Type 1	Type 4	24	10	0.520166215360434	0.6029	0.6699	ns
Threonine	Type 1	Type 5	24	12	2.65500997368517	0.0079	0.0397	*
Threonine	Type 2	Type 3	25	21	-0.74076347575017	0.4588	0.5735	ns
Threonine	Type 2	Type 4	25	10	-1.6460974833417	0.0997	0.1995	ns
Threonine	Type 2	Type 5	25	12	0.361603701729927	0.7176	0.7176	ns
Threonine	Type 3	Type 4	21	10	-1.03235512621702	0.3019	0.4838	ns
Threonine	Type 3	Type 5	21	12	0.956856231981074	0.3386	0.4838	ns
Threonine	Type 4	Type 5	10	12	1.73505070339324	0.0827	0.1995	ns

Abbreviations: FDR: false discovery rate; ns: not significant.
Significance levels: * $p < 0.05$, ** $p < 0.01$

Table S2. Effect size estimates (Cliff s delta) for pairwise comparisons across OCB types.

Metabolite	Group 1	Group 2	n1	n2	Cliff's delta	Effect size magnitude
Leucine	Type 1	Type 2	24	25	-0.53	large
Leucine	Type 1	Type 3	24	21	-0.226190476190476	small
Leucine	Type 1	Type 4	24	10	-0.166666666666667	small
Leucine	Type 1	Type 5	24	12	0.0729166666666667	negligible
Leucine	Type 2	Type 3	25	21	0.401904761904762	medium
Leucine	Type 2	Type 4	25	10	0.38	medium
Leucine	Type 2	Type 5	25	12	0.606666666666667	large
Leucine	Type 3	Type 4	21	10	-0.00476190476190477	negligible
Leucine	Type 3	Type 5	21	12	0.28968253968254	small
Leucine	Type 4	Type 5	10	12	0.316666666666667	small
2-Oxoglutaric acid	Type 1	Type 2	24	25	-0.353333333333333	medium
2-Oxoglutaric acid	Type 1	Type 3	24	21	0.172619047619048	small
2-Oxoglutaric acid	Type 1	Type 4	24	10	0.220833333333333	small

2-Oxoglutaric acid	Type 1	Type 5	24	12	0.46875	medium
2-Oxoglutaric acid	Type 2	Type 3	25	21	0.419047619047619	medium
2-Oxoglutaric acid	Type 2	Type 4	25	10	464	medium
2-Oxoglutaric acid	Type 2	Type 5	25	12	0.686666666666667	large
2-Oxoglutaric acid	Type 3	Type 4	21	10	0.0285714285714286	negligible
2-Oxoglutaric acid	Type 3	Type 5	21	12	0.448412698412698	medium
2-Oxoglutaric acid	Type 4	Type 5	10	12	0.458333333333333	medium
Citric acid	Type 1	Type 2	24	25	-0.236666666666667	small
Citric acid	Type 1	Type 3	24	21	-0.273809523809524	small
Citric acid	Type 1	Type 4	24	10	-0.129166666666667	negligible
Citric acid	Type 1	Type 5	24	12	-0.635416666666667	large
Citric acid	Type 2	Type 3	25	21	-0.0419047619047619	negligible
Citric acid	Type 2	Type 4	25	10	72	negligible

Citric acid	Type 2	Type 5	25	12	-0.396666666666667	medium
Citric acid	Type 3	Type 4	21	10	0.123809523809524	negligible
Citric acid	Type 3	Type 5	21	12	-0.349206349206349	medium
Citric acid	Type 4	Type 5	10	12	-0.391666666666667	medium
Glycerol	Type 1	Type 2	24	25	205	small
Glycerol	Type 1	Type 3	24	21	0.208333333333333	small
Glycerol	Type 1	Type 4	24	10	0.0625	negligible
Glycerol	Type 1	Type 5	24	12	0.583333333333333	large
Glycerol	Type 2	Type 3	25	21	1.99970248693226e-17	negligible
Glycerol	Type 2	Type 4	25	10	-188	small
Glycerol	Type 2	Type 5	25	12	0.286666666666667	small
Glycerol	Type 3	Type 4	21	10	-0.176190476190476	small
Glycerol	Type 3	Type 5	21	12	0.365079365079365	medium
Glycerol	Type 4	Type 5	10	12	0.5	large
Histidine	Type 1	Type 2	24	25	-485	large

Histidine	Type 1	Type 3	24	21	-0.158730158730159	small
Histidine	Type 1	Type 4	24	10	-0.354166666666667	medium
Histidine	Type 1	Type 5	24	12	0.0486111111111111	negligible
Histidine	Type 2	Type 3	25	21	0.382857142857143	medium
Histidine	Type 2	Type 4	25	10	0.24	small
Histidine	Type 2	Type 5	25	12	0.53	large
Histidine	Type 3	Type 4	21	10	-0.171428571428571	small
Histidine	Type 3	Type 5	21	12	0.234126984126984	small
Histidine	Type 4	Type 5	10	12	0.366666666666667	medium
Threonine	Type 1	Type 2	24	25	-455	medium
Threonine	Type 1	Type 3	24	21	-0.307539682539683	small
Threonine	Type 1	Type 4	24	10	-0.166666666666667	small
Threonine	Type 1	Type 5	24	12	-0.444444444444444	medium
Threonine	Type 2	Type 3	25	21	0.108571428571429	negligible
Threonine	Type 2	Type 4	25	10	0.38	medium

Threonine	Type 2	Type 5	25	12	-0.123333333333333	negligible
Threonine	Type 3	Type 4	21	10	0.20952380952381	small
Threonine	Type 3	Type 5	21	12	-0.178571428571429	small
Threonine	Type 4	Type 5	10	12	-0.433333333333333	medium

Effect size interpretation: negligible (<0.147); small (0.147–0.33); medium (0.33–0.474); large (>0.474)

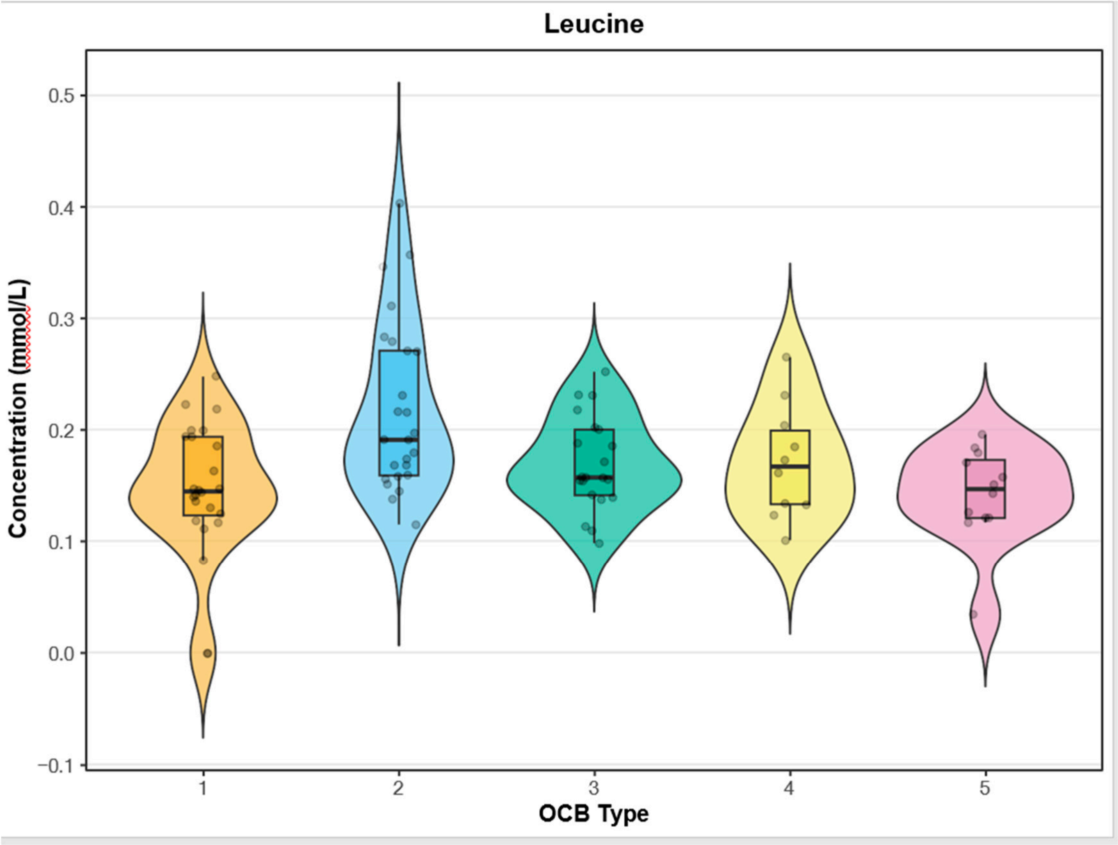
Leucine
Concentration (mmol/L)
0.5
0.4
0.3
0.2
0.1
0.0
−0.1
1
2
3
4
5
OCB Type

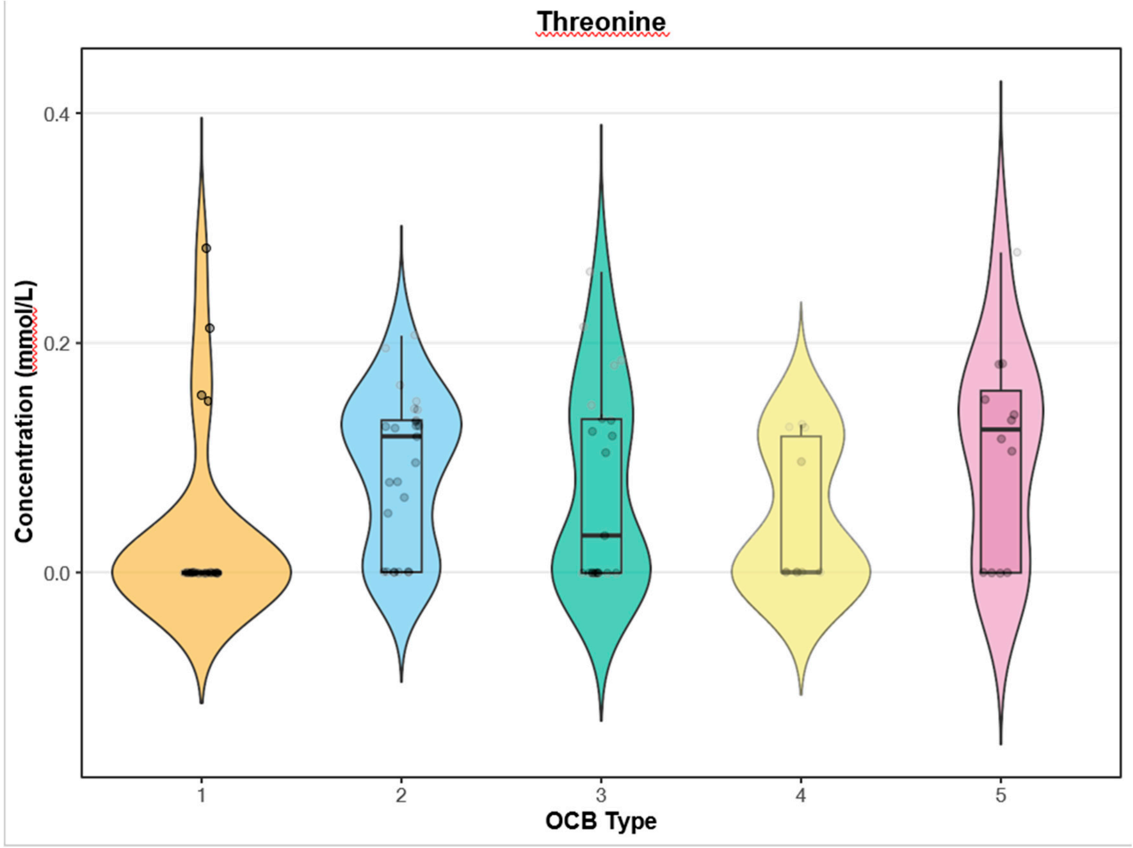
Threonine
Concentration (mmol/L)
0.4
0.2
0.0
1
2
3
4
5
OCB Type

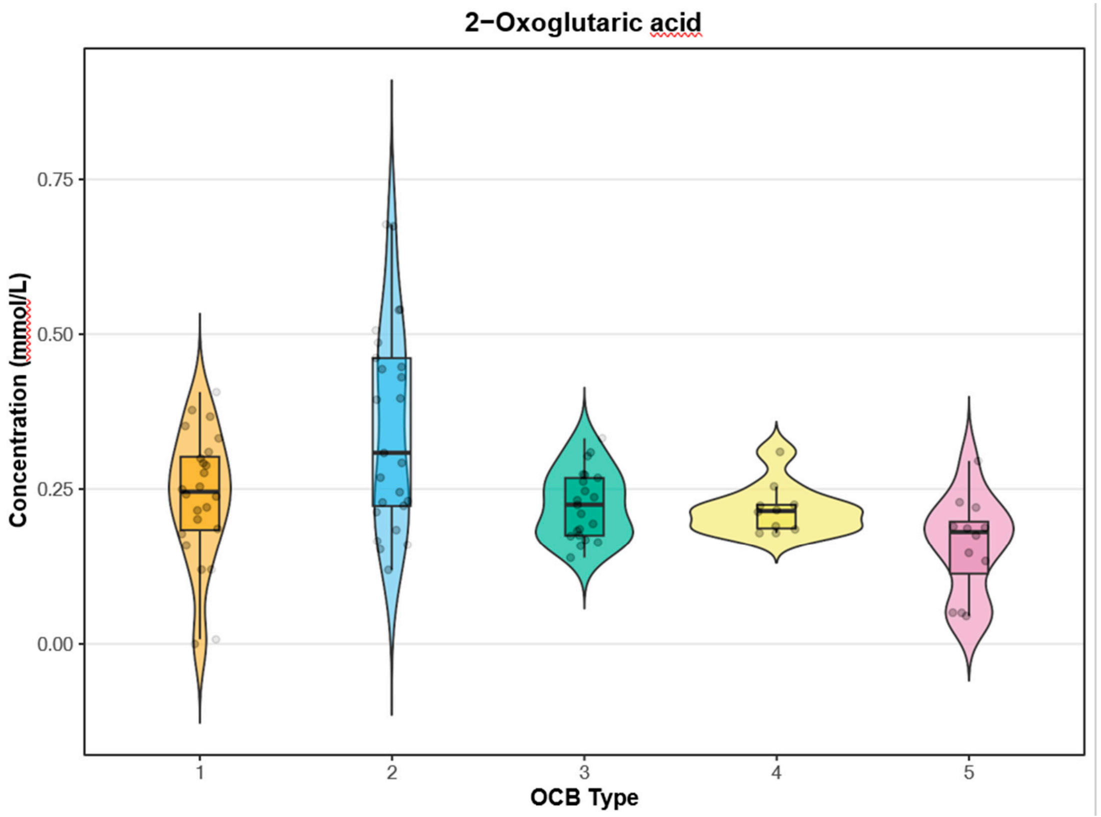

2−Oxoglutaric acid
Concentration (mmol/L)
0.75
0.50
0.25
0.00
1
2
3
4
5
OCB Type

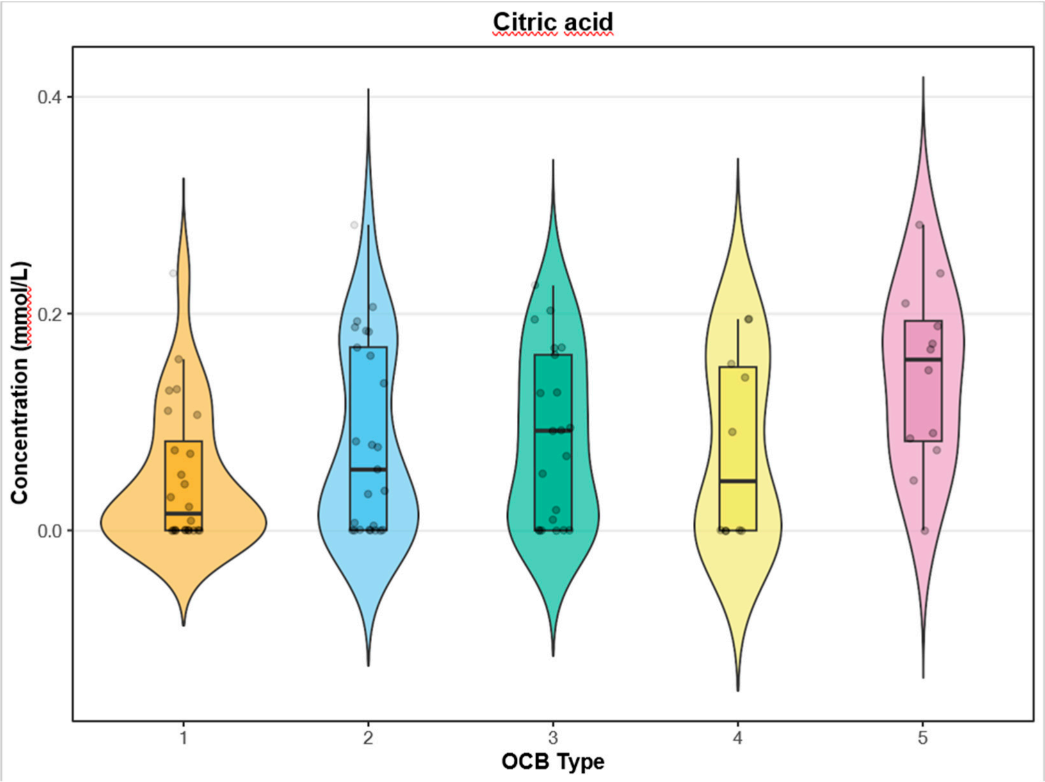
Citric acid
Concentration (mmol/L)
0.4
0.2
0.0
1
2
3
4
5
OCB Type

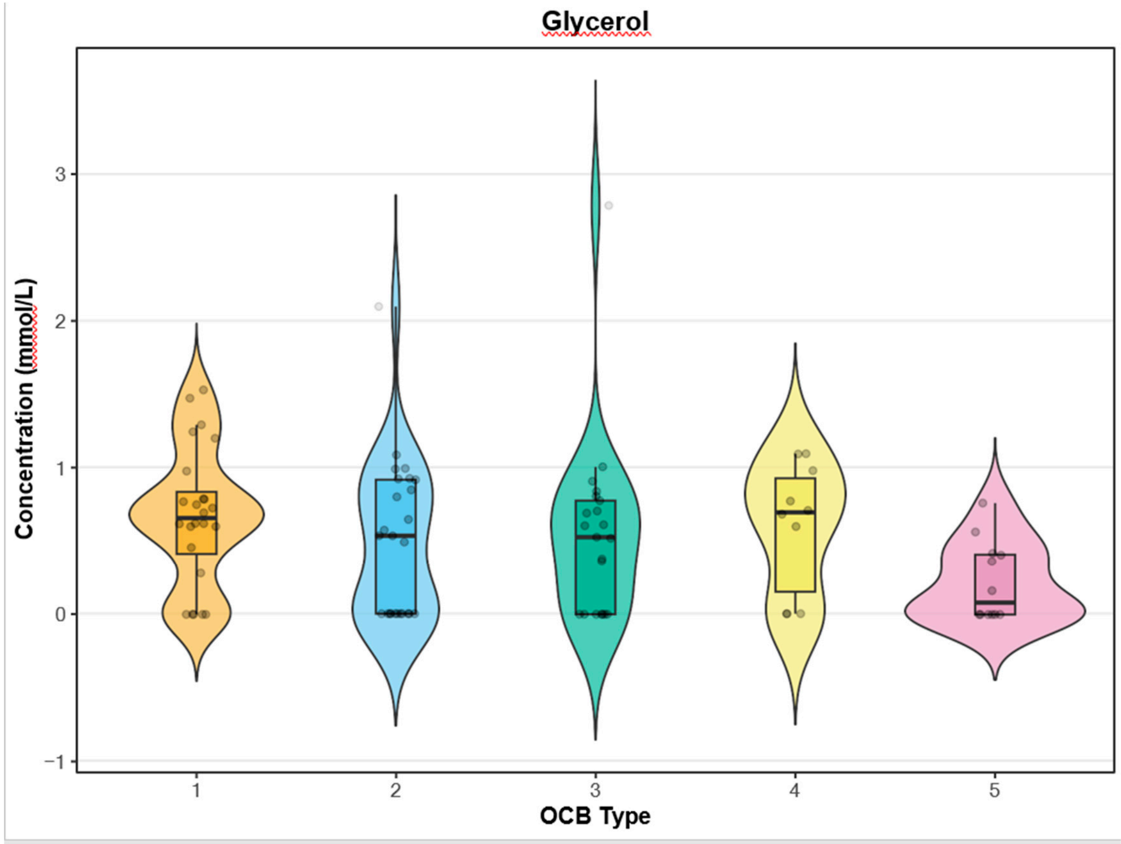

Glycerol
Concentration (mmol/L)
3
2
1
0
−1
1
2
3
4
5
OCB Type

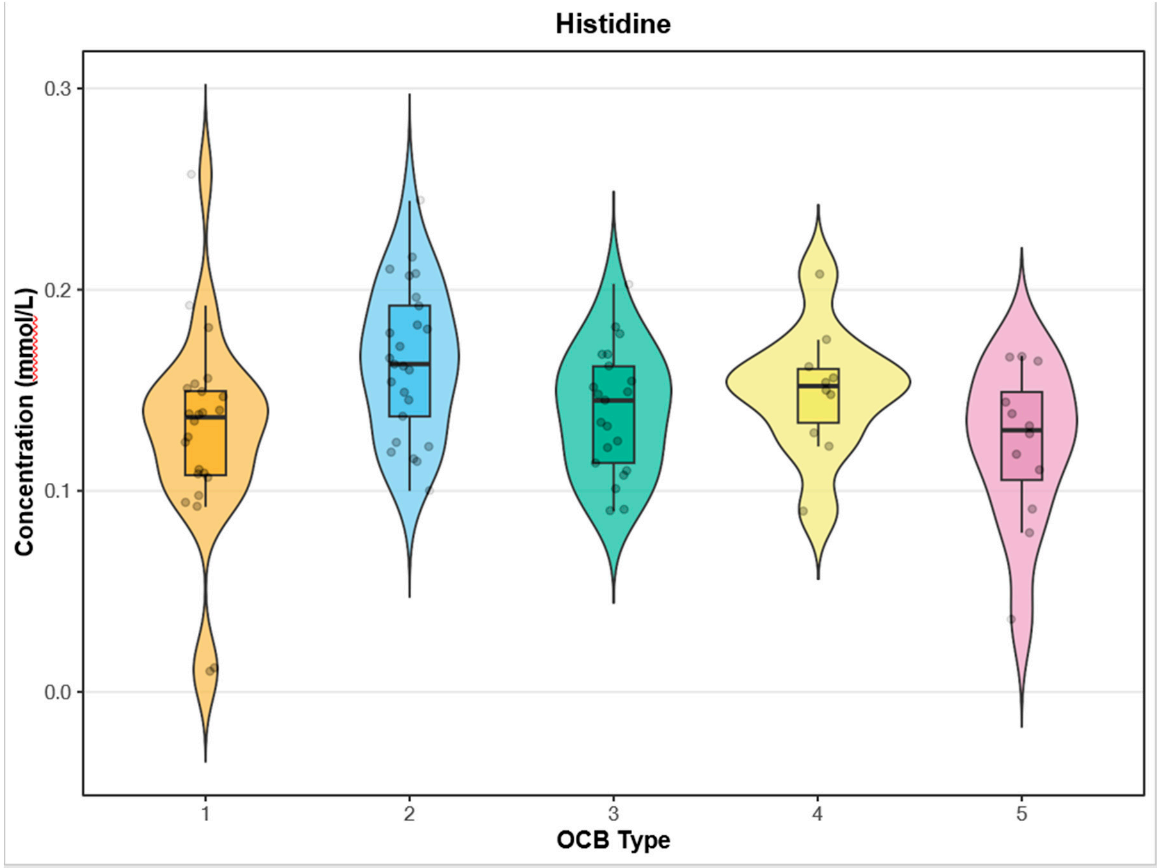

Histidine
0.3
0.2
0.1
0.0
Concentration (mmol/L)
1
2
3
4
5
OCB Type

Figure S1 A-F. Distribution of selected serum metabolites across OCB types.
(A) Leucine, (B) threonine, (C) 2-oxoglutaric acid, (D) citric acid, (E) glycerol, and (F) histidine concentrations (mmol/L) across OCB Types 1–5.
Violin plots illustrate the full distribution of the data; embedded boxplots indicate median and interquartile range, and points represent individual samples.

Figure S2A

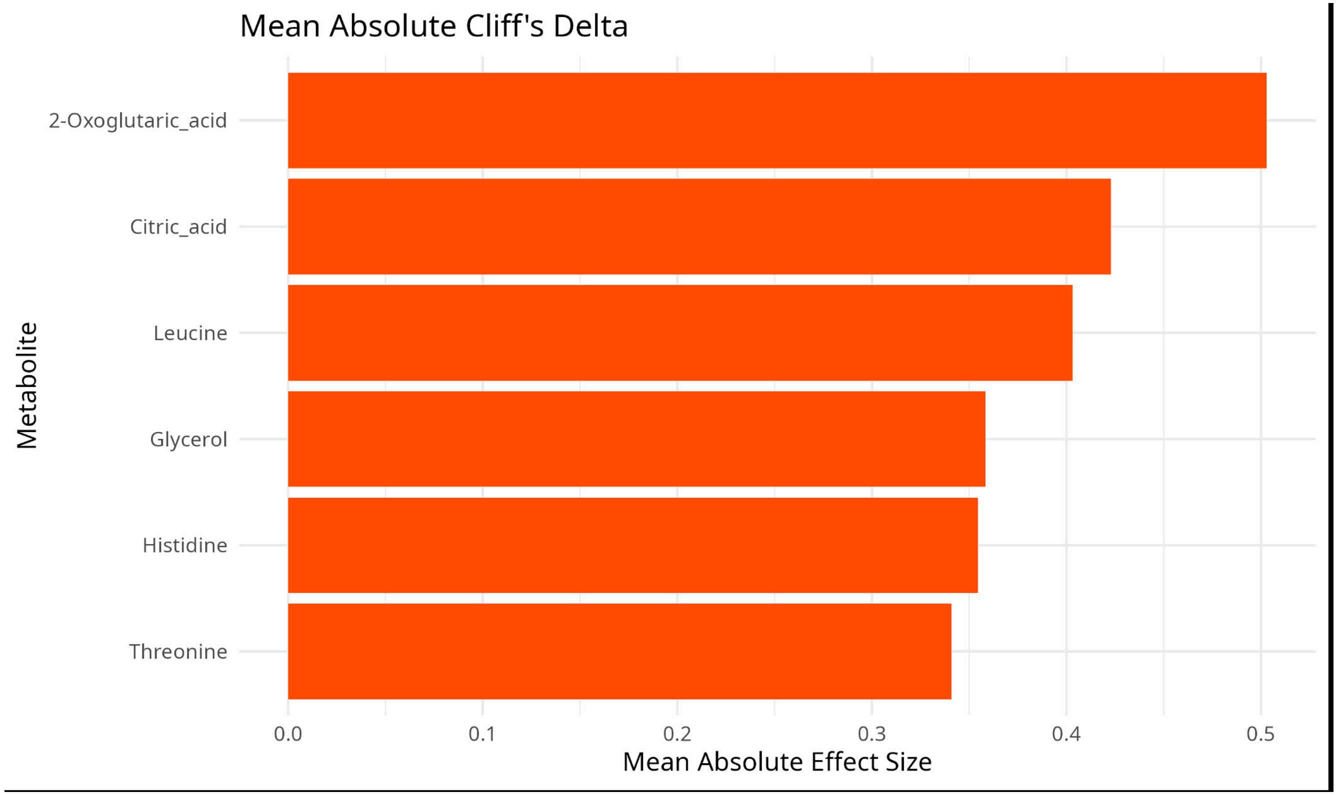

Mean Absolute Cliff's Delta
2-Oxoglutaric_acid
Citric_acid
Leucine
Glycerol
Histidine
Threonine
Metabolite
0.0
0.1
0.2
0.3
0.4
0.5
Mean Absolute Effect Size

Figure S2B

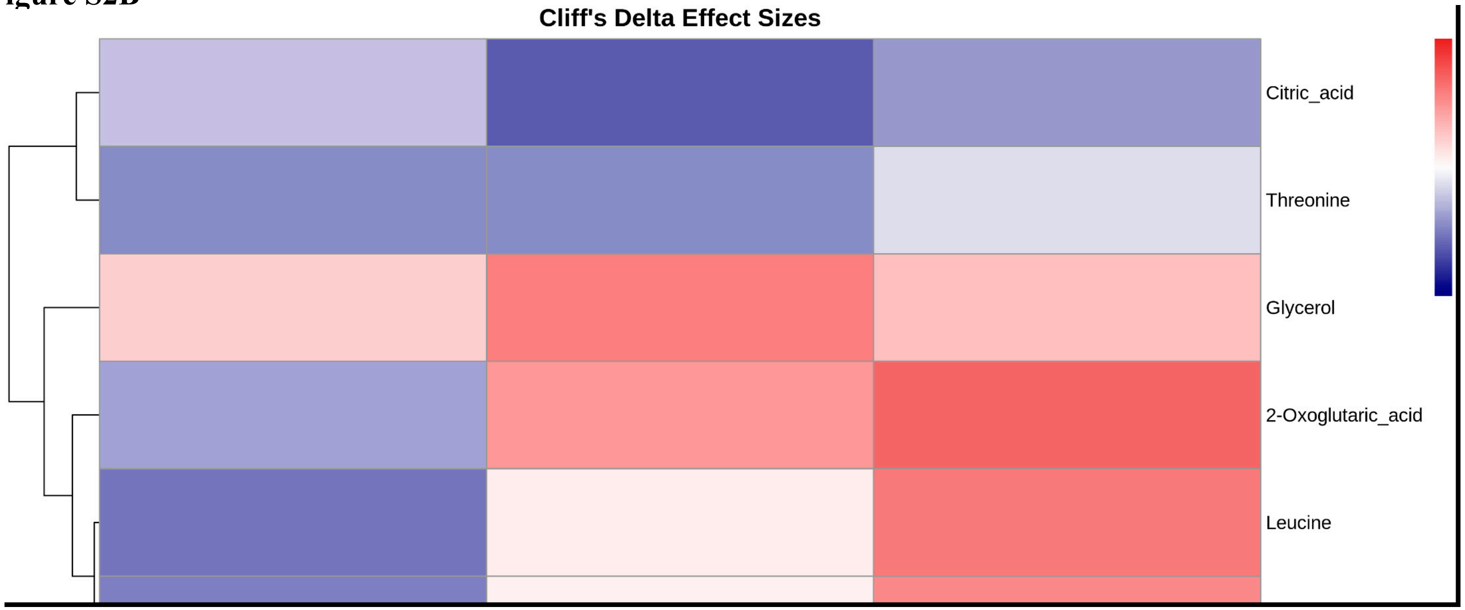

Figure S2. Effect size patterns across metabolites and OCB types.

(A) Mean absolute Cliff's delta values summarising the magnitude of group differences across metabolites.

(B) Heatmap of pairwise Cliff's delta effect sizes illustrating the direction and magnitude of differences between OCB subgroups. Warmer colours indicate higher values, while cooler colours indicate lower values.

Figure S3

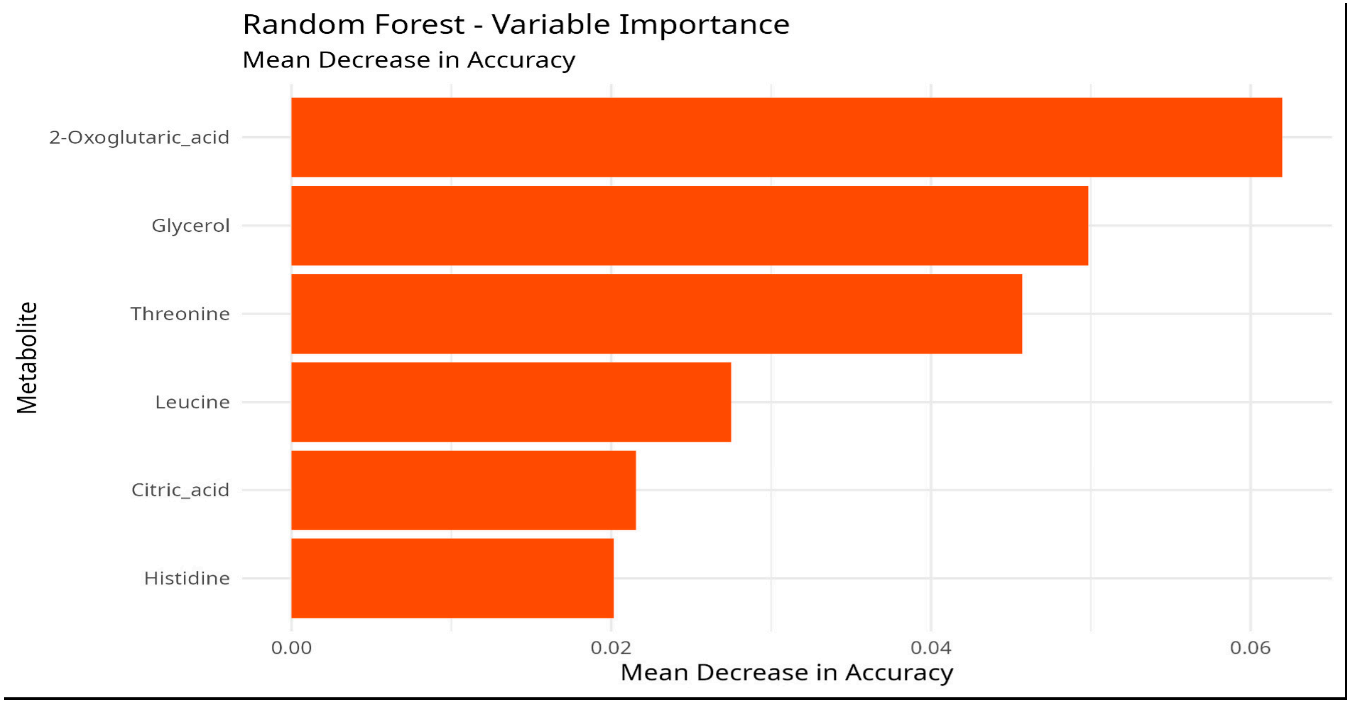

Figure S3. Random forest variable importance analysis of serum metabolites across OCB types. Variable importance is expressed as mean decrease in accuracy, indicating the contribution of each metabolite to classification performance. Higher values reflect greater importance in distinguishing between OCB subgroups.

Figure S4

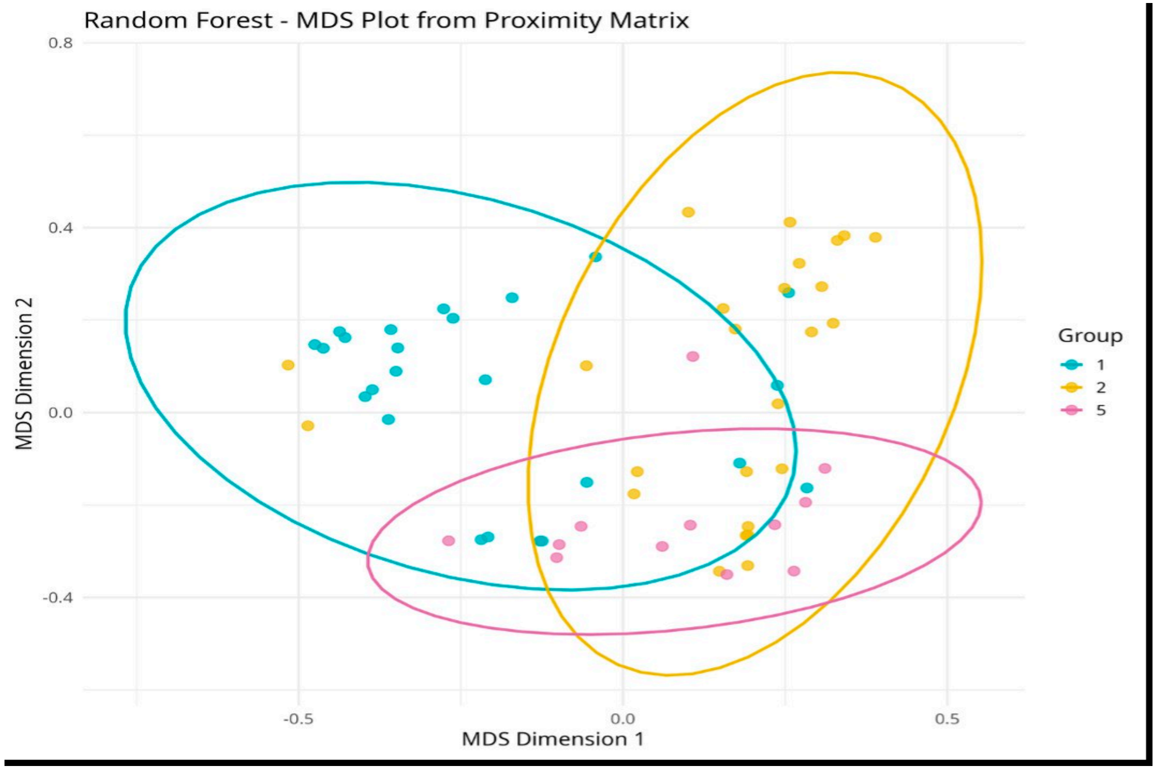

Figure S4. Random forest multidimensional scaling (MDS) plot derived from the proximity matrix. Samples are projected into a two-dimensional space representing similarity in classification patterns. Points are coloured by OCB group, and ellipses indicate group dispersion. The observed overlap between groups is consistent with limited classification separation and supports the exploratory nature of the model.

FIGURE S4
A.

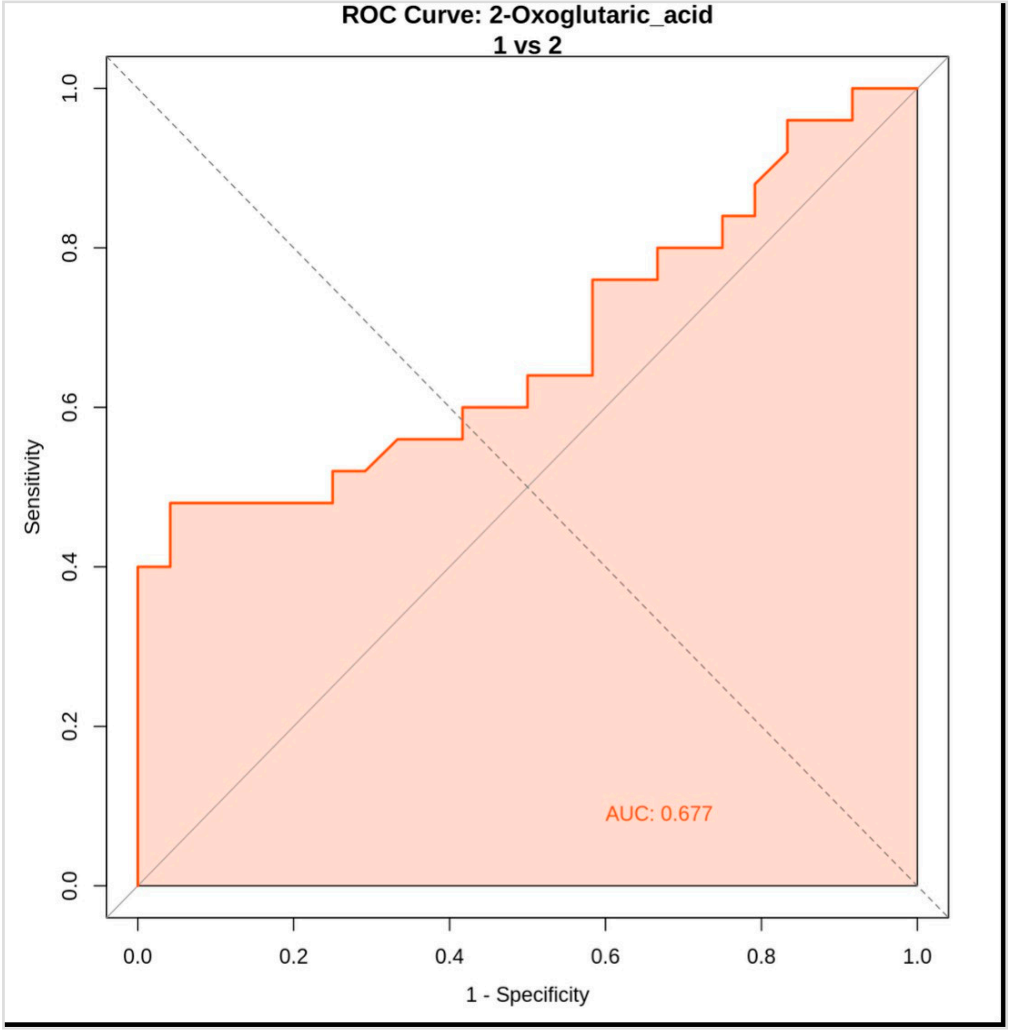
ROC Curve: 2-Oxoglutaric_acid
1 vs 2
Sensitivity
1 - Specificity
0.0
0.2
0.4
0.6
0.8
1.0
AUC: 0.677

B.

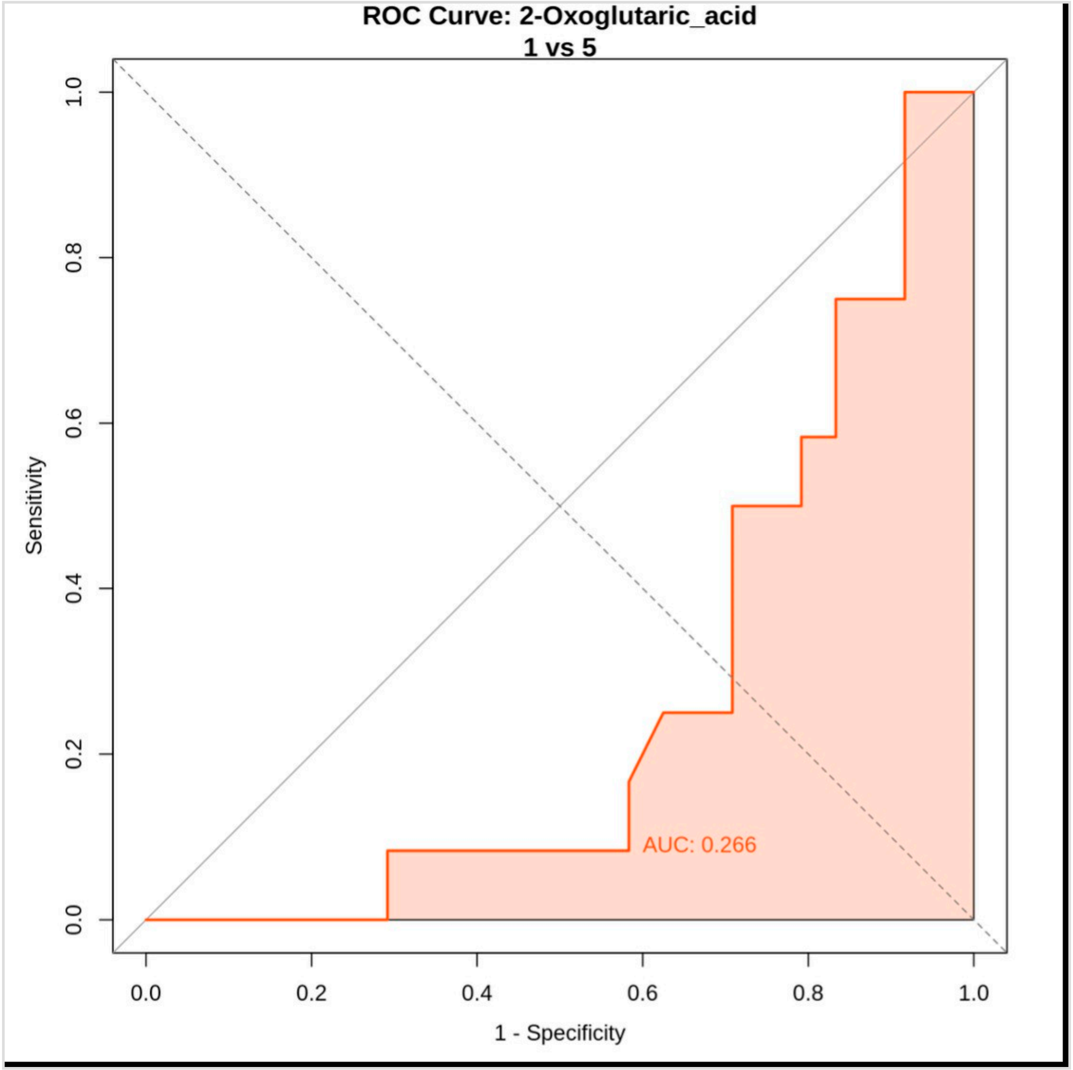

ROC Curve: 2-Oxoglutaric_acid
1 vs 5
Sensitivity
1 - Specificity
AUC: 0.266
0.0
0.2
0.4
0.6
0.8
1.0

C.

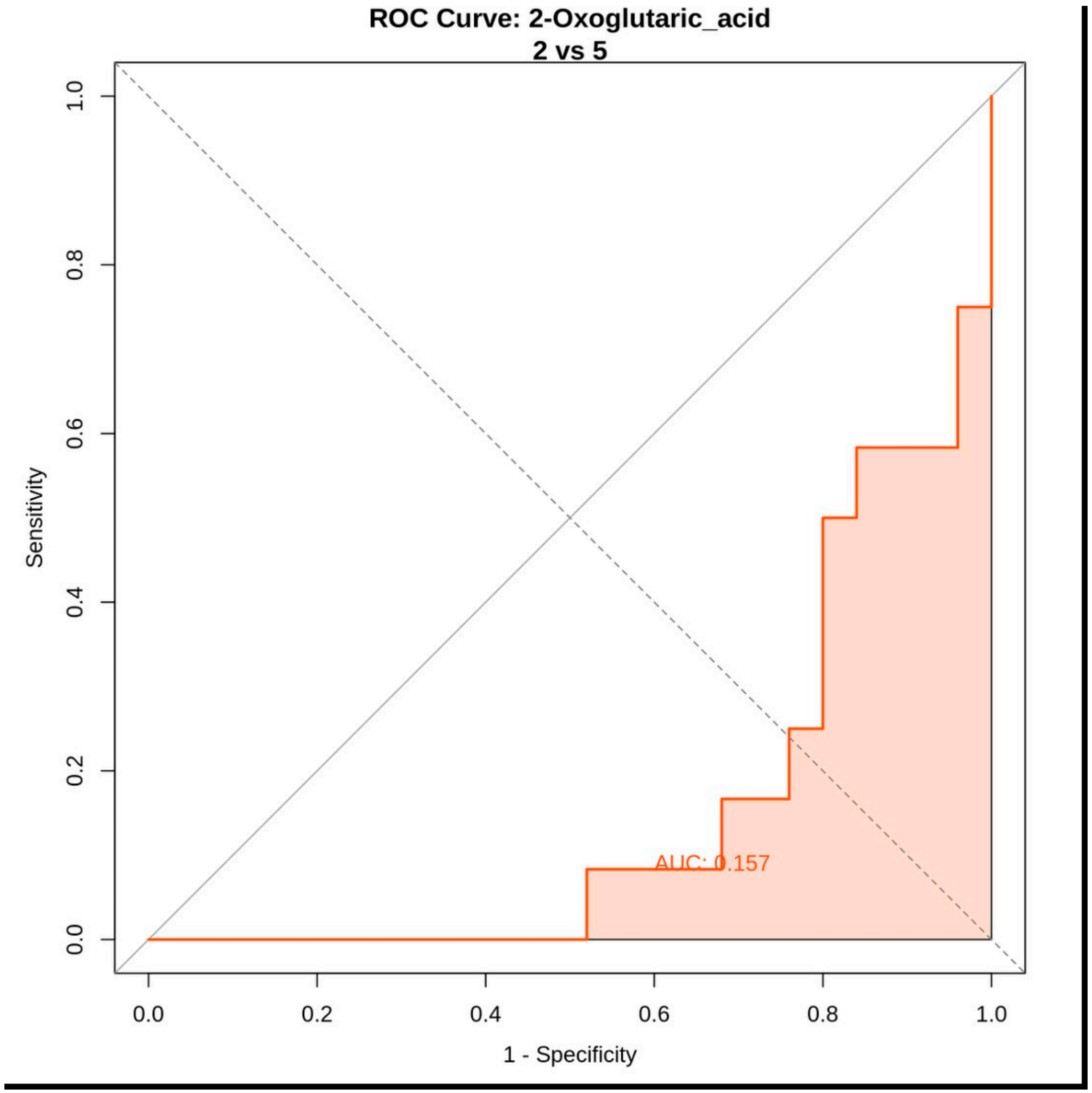
ROC Curve: 2-Oxoglutaric_acid
2 vs 5
Sensitivity
0.0
0.2
0.4
0.6
0.8
1.0
1 - Specificity
AUC: 0.157

D.

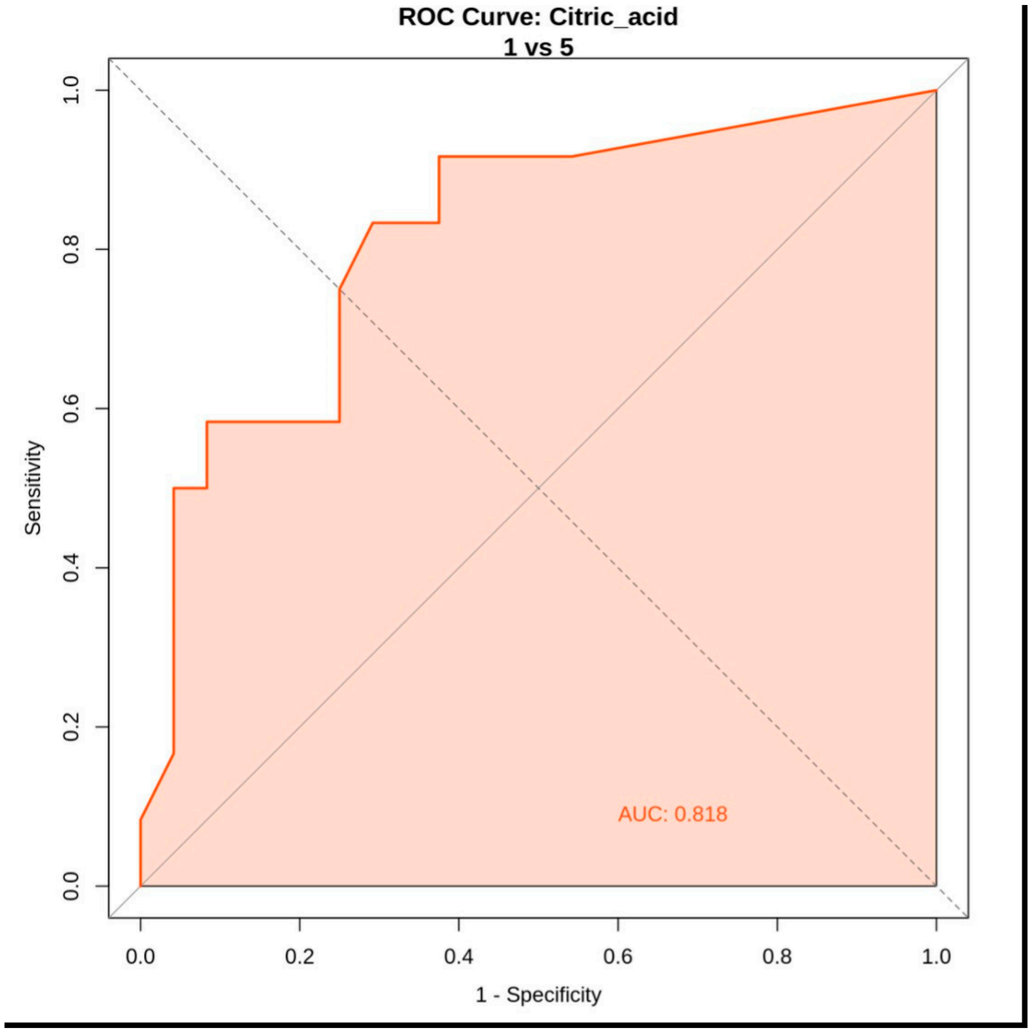

ROC Curve: Citric_acid
1 vs 5
Sensitivity
1 - Specificity
0.0
0.2
0.4
0.6
0.8
1.0
AUC: 0.818

E.

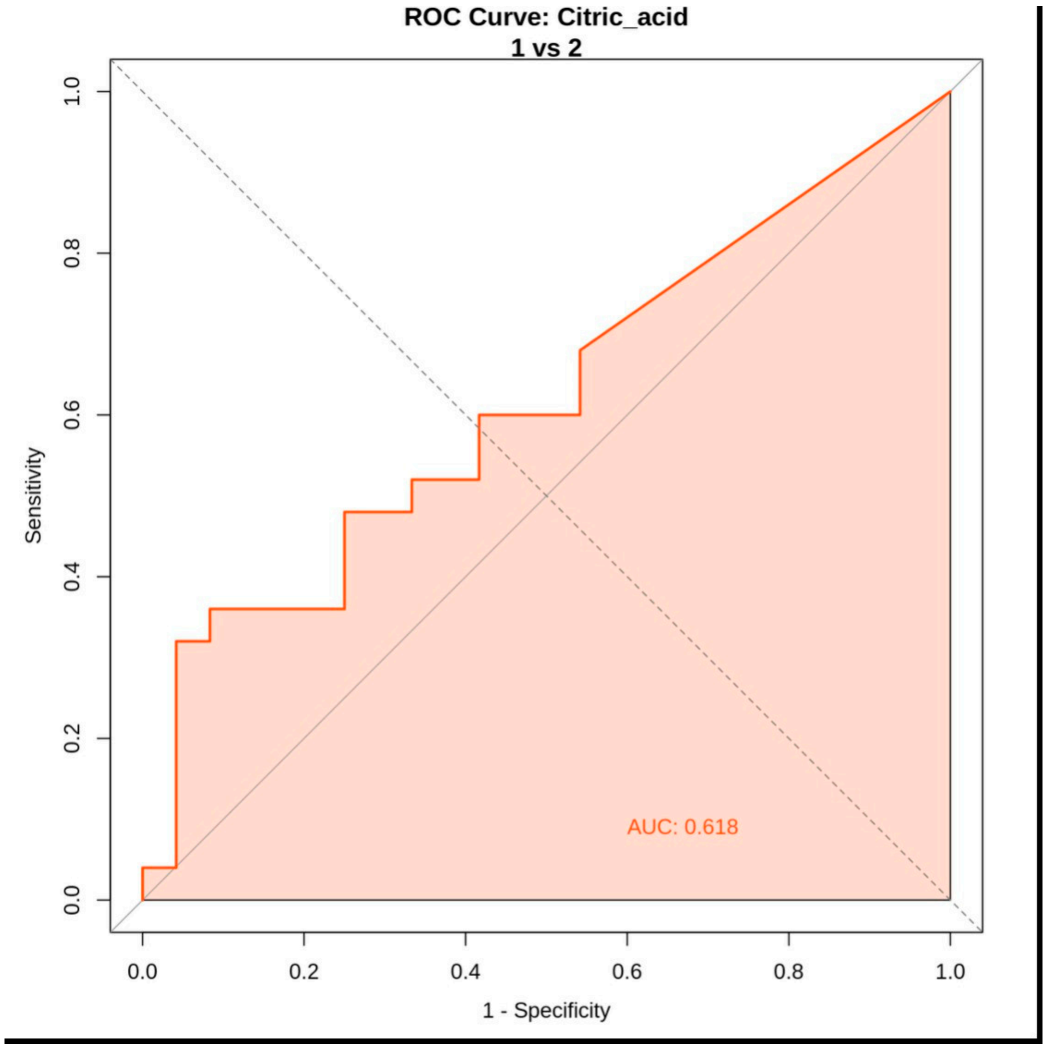

ROC Curve: Citric_acid
1 vs 2
Sensitivity
1 - Specificity
0.0
0.2
0.4
0.6
0.8
1.0
AUC: 0.618

F.

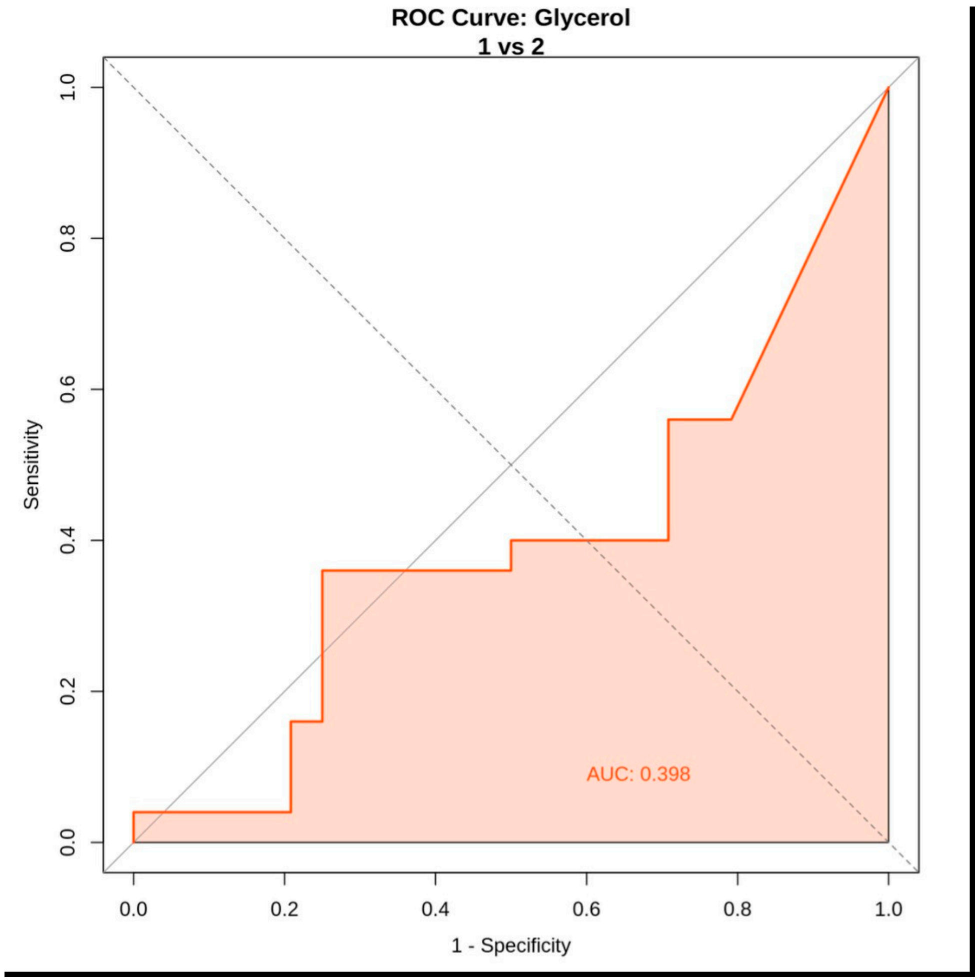

ROC Curve: Glycerol
1 vs 2
Sensitivity
1 - Specificity
AUC: 0.398
0.0
0.2
0.4
0.6
0.8
1.0

G.

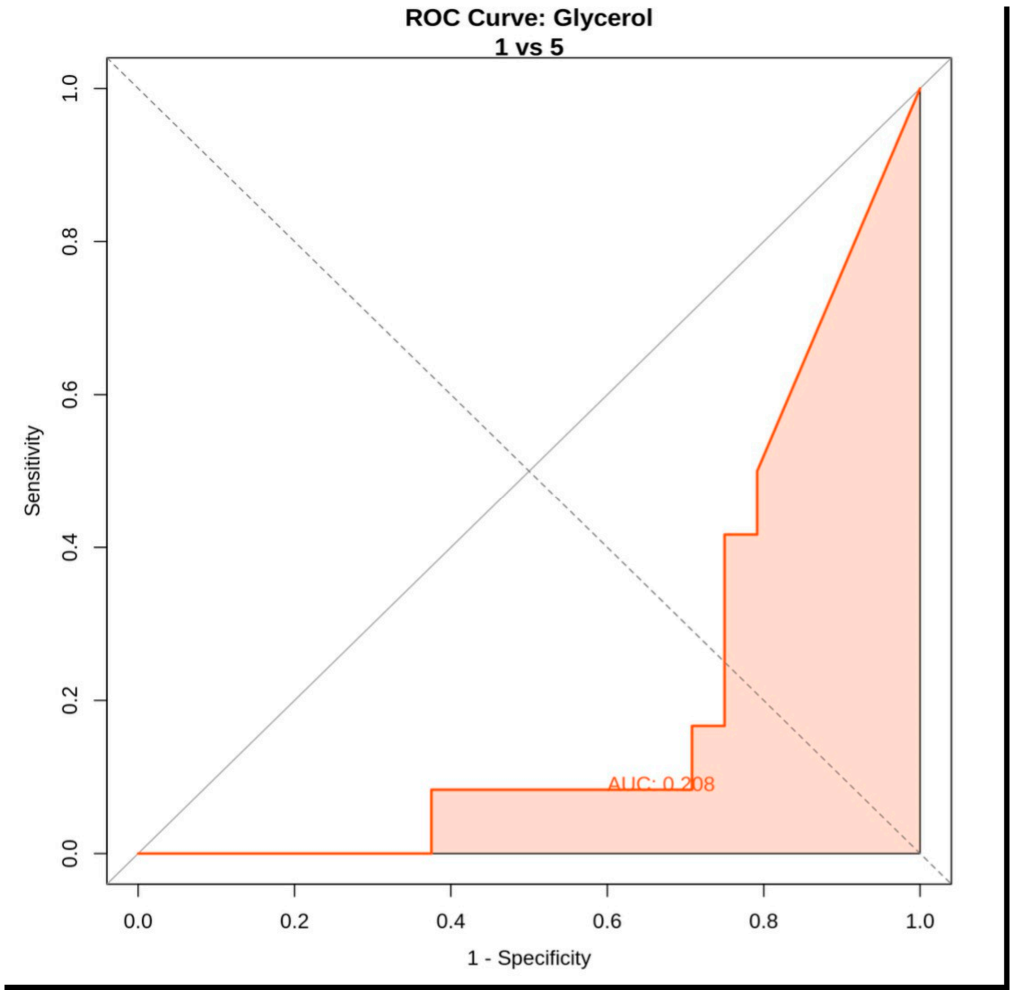

Figure S4. Receiver operating characteristic (ROC) curves illustrating the discriminative performance of selected metabolites across pairwise OCB group comparisons.

Figure S5

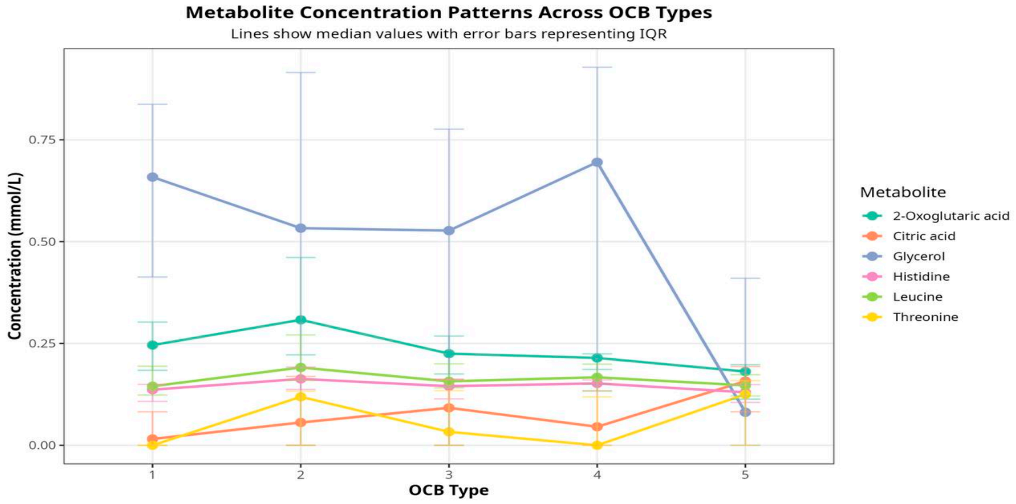

Figure S5. Median concentration trends of serum metabolites across OCB types.
Line plots showing median metabolite concentrations with interquartile range (IQR) error bars across OCB Types 1–5. This figure provides a complementary summary view of distributional patterns observed in the violin plots (Figure S1), highlighting overall trends and variability across metabolites.

Figure S5

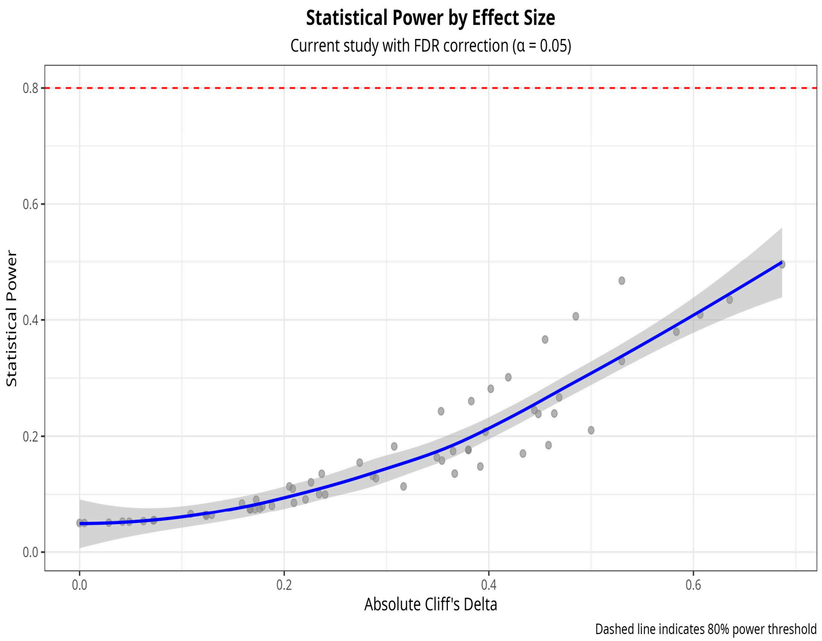

Figure S5. Statistical power plotted against effect size (absolute Cliff's delta) under FDR correction (α = 0.05). Points correspond to individual pairwise comparisons, with a fitted trend line and confidence interval. The dashed horizontal line indicates the 80% power threshold. The analysis highlights insufficient statistical power to reliably detect small and moderate effect sizes in the current dataset.

Figure S6

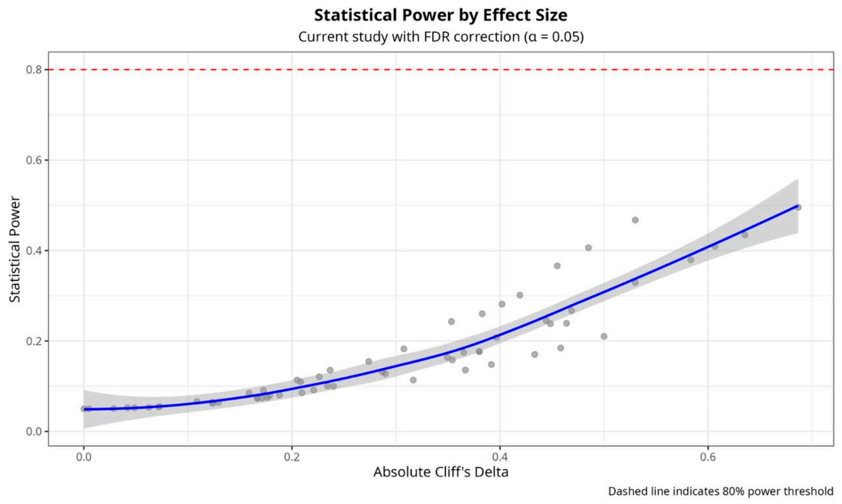

Figure S6. Statistical power as a function of effect size.
Relationship between absolute effect size (Cliff's delta) and statistical power under the current study design with false discovery rate (FDR) correction. The dashed line indicates the conventional 80% power threshold, demonstrating that the present study is underpowered to detect small-to-moderate effect sizes.

Figure S6

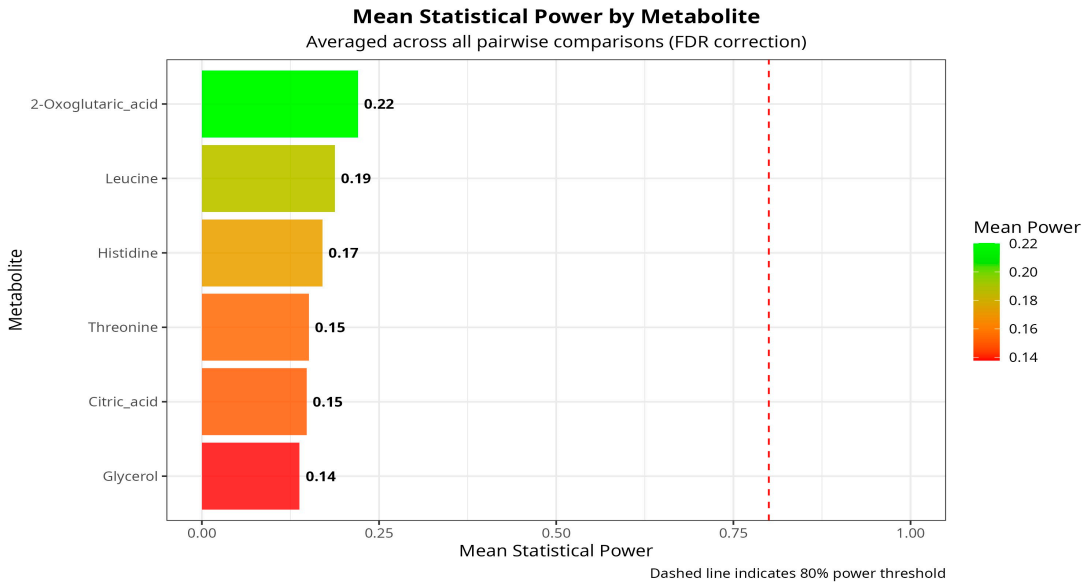

Figure S6. Mean statistical power estimates for each metabolite across all pairwise comparisons under FDR correction.

Values represent the average power derived from pairwise tests, with the dashed vertical line indicating the 80% threshold for adequate statistical power. The consistently low power across metabolites highlights insufficient sample size to reliably detect small-to-moderate effects.

Figure S7

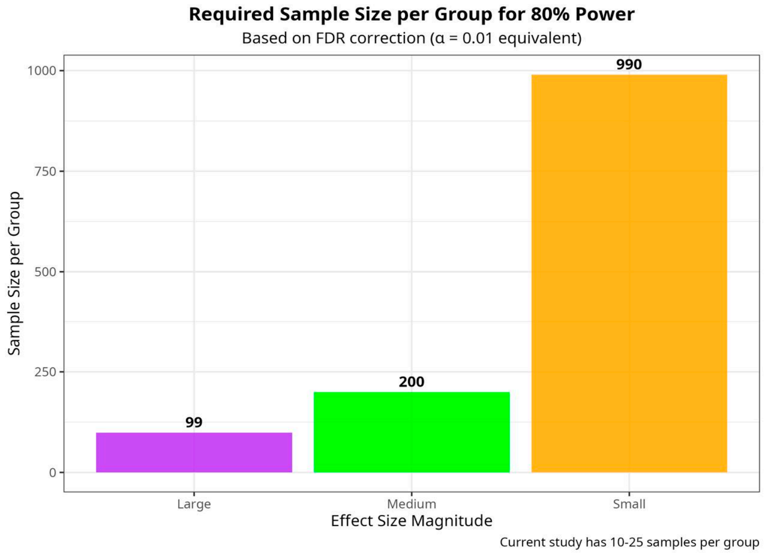

Figure S7. Required sample size per group for achieving 80% statistical power.
Estimated sample sizes required to achieve 80% power across different effect size magnitudes under FDR correction. The current study sample sizes (approximately 10–25 per group) are shown to be sufficient for large effects but underpowered for detecting small and moderate effects.

Figure S8

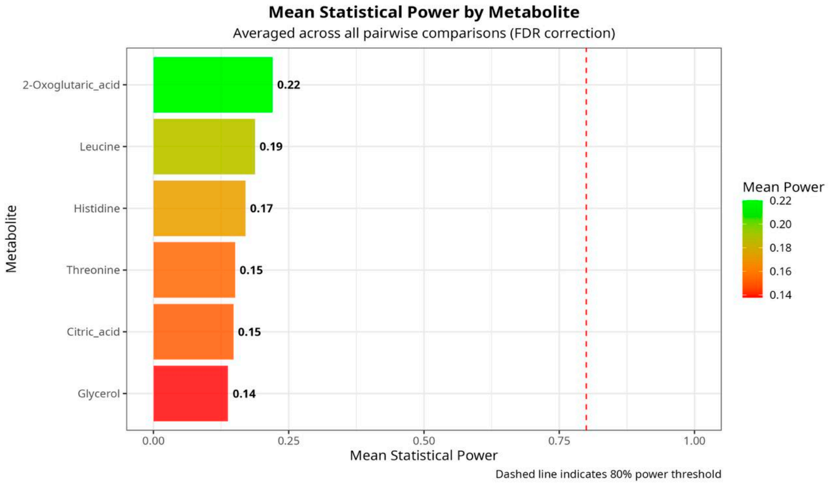

Figure S8. Mean statistical power across metabolites.
Average statistical power estimates calculated across all pairwise comparisons for each metabolite under FDR correction. The dashed line represents the 80% power threshold, highlighting variability in detectability across metabolites and overall limited power in the current dataset.
